# Supplementary material for: A ceratopsid-dominated tracksite from the Dinosaur Park Formation (Campanian) at Dinosaur Provincial Park, Alberta, Canada
Source: PLoS One. 2025 Jul 23;20(7):e0324913. doi: 10.1371/journal.pone.0324913 (PMC12286367; doi:10.1371/journal.pone.0324913)
Supplement: S1 Models — https://mq.pedestal3d.com/r/bePfWqNGay/. (DOCX) [file pone.0324913.s001.docx]

**A ceratopsid-dominated tracksite from the Dinosaur Park Formation (Campanian) at Dinosaur Provincial Park, Alberta, Canada**

Phil R. Bell^1¶^, Brian J. Pickles^2¶*^, Sarah C. Ashby^2^, Issy E. Walker^2^, Sally Hurst^3^, Michael Rampe^4^, Paul Durkin^5^, Caleb M. Brown^5,6,7^

^1^Palaeoscience Research Centre, School of Environmental and Rural Science, University of New England, Armidale, NSW, Australia.

^2^School of Biological Sciences, University of Reading, Whiteknights, Reading, UK.

^3^School of Natural Sciences, Macquarie University, Macquarie Park, NSW, Australia.

^4^Faculty of Arts, Macquarie University, Macquarie Park, NSW, Australia.

^5^University of Manitoba, Department of Earth Sciences, Winnipeg, Manitoba, Canada.

^6^Royal Tyrrell Museum of Palaeontology, Drumheller, Alberta, Canada.

^7^University of Alberta, Department of Biological Sciences, Edmonton, Alberta, Canada.

*Corresponding author:

E-mail: [b.j.pickles@reading.ac.uk](mailto:b.j.pickles@reading.ac.uk) (BJP)

^¶^These authors contributed equally to this work.

**Supporting Information**

**S1 Models.** 3D Model Gallery displaying: i) the Skyline Tracksite following the initial 3-day excavation on 22^nd^ July 2024, ii) individual tracks (C1.1, A1.1, T1.2, T2.1; taken 22^nd^ July 2024), and iii) position of C1.1 and T1.2 in context (taken 22^nd^ August 2024) <https://mq.pedestal3d.com/r/bePfWqNGay/>
