# Supplementary material for: A ceratopsid-dominated tracksite from the Dinosaur Park Formation (Campanian) at Dinosaur Provincial Park, Alberta, Canada
Source: PLoS One. 2025 Jul 23;20(7):e0324913. doi: 10.1371/journal.pone.0324913 (PMC12286367; doi:10.1371/journal.pone.0324913)

PLOS ONE

A ceratopsid-dominated tracksite from the Dinosaur Park Formation (Campanian) at Dinosaur Provincial Park, Alberta, Canada

--Manuscript Draft--

**Manuscript Number:**	PONE-D-25-02384
**Article Type:**	Research Article
**Full Title:**	A ceratopsid-dominated tracksite from the Dinosaur Park Formation (Campanian) at Dinosaur Provincial Park, Alberta, Canada
**Short Title:**	A ceratopsid-dominated tracksite from the Dinosaur Park Formation
**Corresponding Author:**	Brian John Pickles, Ph.D.<br>University of Reading<br>Reading, UNITED KINGDOM OF GREAT BRITAIN AND NORTHERN IRELAND
**Keywords:**	Ankylosauria; Ceratopsia; Dinosaur Park Formation; ichnology; palaeoecology; Theropoda; Tyrannosauridae.
**Abstract:**	The badlands of Dinosaur Provincial Park (Alberta, Canada) are renowned for the exceptional abundance and diversity of Campanian-aged vertebrate body fossils, especially dinosaurs. Due to the steep exposures and rapid erosion, dinosaur tracks and trackways are considered extremely rare but have been recorded from a small number of concretionary casts, which pertain to hadrosaurids and a single tyrannosaurid. Here, we document the first multitaxic dinosaur footprint assemblage from the Dinosaur Park Formation based on a new locality that contains multiple individual ceratopsids, two tyrannosaurids, a possible ankylosaurian, and a small theropod-like taxon. Ceratopsid tracks are globally rare but dominate the new tracksite, suggesting gregarious behaviour, which is also supported by their regular spacing and parallel arrangement. The possible ankylosaurian track is identified (in part) on account of having three distinct pedal digits, consistent with the pedal anatomy of several Dinosaur Park ankylosaurids (<i>Euoplocephalus</i>, <i>Dyoplosaurus</i>) but differentiating it from previously-reported ankylosaurian tracks (<i>Tetrapodosaurus</i> isp.). Importantly, the new tracks are the first natural moulds (concave epirelief) found in Dinosaur Provincial Park, which, due to the unique geomorphology of the area, can only be recognised in outcrops where there are prominent sediment displacement rims. The new search image outlined here has already resulted in several subsequent trackway discoveries, and has the potential to transform ichnological studies in the Dinosaur Park Formation and related formations where badlands prevail.
**Order of Authors:**	Phil R. Bell, Ph.D.
	Brian J. Pickles, Ph.D.
	Sarah C. Ashby, BSc
	Issy E. Walker, BSc
	Sally Hurst, MRes
	Michael Rampe
	Paul Durkin, Ph.D.
	Caleb M. Brown, Ph.D.
**Additional Information:**	
**Question**	**Response**
**Financial Disclosure**<br><br>Enter a financial disclosure statement that describes the sources of funding for the work included in this submission. Review the submission guidelines for detailed	Yes

Powered by Editorial Manager® and ProduXion Manager® from Aries Systems Corporation

requirements. View published research articles from *PLOS ONE* for specific examples.<br><br>This statement is required for submission and **will appear in the published article** if the submission is accepted. Please make sure it is accurate.<br><br>**Funded studies**<br>Enter a statement with the following details:<br>• Initials of the authors who received each award<br>• Grant numbers awarded to each author<br>• The full name of each funder<br>• URL of each funder website<br>• Did the sponsors or funders play any role in the study design, data collection and analysis, decision to publish, or preparation of the manuscript?<br><br>Did you receive funding for this work?	
Please add funding details.<br>as follow-up to "**Financial Disclosure**<br><br>Enter a financial disclosure statement that describes the sources of funding for the work included in this submission. Review the submission guidelines for detailed requirements. View published research articles from *PLOS ONE* for specific examples.<br><br>This statement is required for submission and **will appear in the published article** if the submission is accepted. Please make sure it is accurate.	BJP and PRB received a University of New England Visiting Research Fellowship.<br>There is no Grant Number associated with this award.<br>The funders were not involved in the study design, data collection and analysis, decision to publish, or preparation of the manuscript.

Powered by Editorial Manager® and ProduXion Manager® from Aries Systems Corporation

**Funded studies**<br>Enter a statement with the following details:<br>• Initials of the authors who received each award<br>• Grant numbers awarded to each author<br>• The full name of each funder<br>• URL of each funder website<br>• Did the sponsors or funders play any role in the study design, data collection and analysis, decision to publish, or preparation of the manuscript?<br><br>Did you receive funding for this work?"	
Please select the country of your main research funder (please select carefully as in some cases this is used in fee calculation).<br>as follow-up to "**Financial Disclosure**<br><br>Enter a financial disclosure statement that describes the sources of funding for the work included in this submission. Review the submission guidelines for detailed requirements. View published research articles from *PLOS ONE* for specific examples.<br><br>This statement is required for submission and **will appear in the published article** if the submission is accepted. Please make sure it is accurate.<br><br>**Funded studies**<br>Enter a statement with the following details:<br>• Initials of the authors who received each award<br>• Grant numbers awarded to each author<br>• The full name of each funder<br>• URL of each funder website<br>• Did the sponsors or funders play any role in the study design, data collection and analysis, decision to publish, or preparation of the manuscript?<br><br>Did you receive funding for this work?"	AUSTRALIA - AU
**Competing Interests**	The authors have declared that no competing interests exist.

Powered by Editorial Manager® and ProduXion Manager® from Aries Systems Corporation

Use the instructions below to enter a competing interest statement for this submission. On behalf of all authors, disclose any competing interests that could be perceived to bias this work—acknowledging all financial support and any other relevant financial or non-financial competing interests.<br><br>This statement is required for submission and **will appear in the published article** if the submission is accepted. Please make sure it is accurate and that any funding sources listed in your Funding Information later in the submission form are also declared in your Financial Disclosure statement.<br><br>View published research articles from *PLOS ONE* for specific examples.<br><br>**NO authors have competing interests**<br><br>Enter: *The authors have declared that no competing interests exist.*<br><br>**Authors with competing interests**<br><br>Enter competing interest details beginning with this statement:<br><br>*I have read the journal's policy and the authors of this manuscript have the following competing interests: [insert competing interests here]*<br><br>* typeset	
**Ethics Statement**<br><br>Enter an ethics statement for this submission. This statement is required if the study involved:<br><br>• Human participants<br>• Human specimens or tissue<br>• Vertebrate animals or cephalopods<br>• Vertebrate embryos or tissues	Field Research<br>Fieldwork was conducted under Research and Collection Permit 24-409 (Alberta Parks) and Permit to Excavate Palaeontological Resources 24-047 (Alberta Culture).

Powered by Editorial Manager® and ProduXion Manager® from Aries Systems Corporation

• Field research<br><br>Write "N/A" if the submission does not require an ethics statement.<br><br>General guidance is provided below. Consult the submission guidelines for detailed instructions. **Make sure that all information entered here is included in the Methods section of the manuscript.**	

Powered by Editorial Manager® and ProduXion Manager® from Aries Systems Corporation

**Format for specific study types**<br><br>**Human Subject Research (involving human participants and/or tissue)**<br>• Give the name of the institutional review board or ethics committee that approved the study<br>• Include the approval number and/or a statement indicating approval of this research<br>• Indicate the form of consent obtained (written/oral) or the reason that consent was not obtained (e.g. the data were analyzed anonymously)<br><br>**Animal Research (involving vertebrate animals, embryos or tissues)**<br>• Provide the name of the Institutional Animal Care and Use Committee (IACUC) or other relevant ethics board that reviewed the study protocol, and indicate whether they approved this research or granted a formal waiver of ethical approval<br>• Include an approval number if one was obtained<br>• If the study involved *non-human primates*, add *additional details* about animal welfare and steps taken to ameliorate suffering<br>• If anesthesia, euthanasia, or any kind of animal sacrifice is part of the study, include briefly which substances and/or methods were applied<br><br>**Field Research**<br><br>Include the following details if this study involves the collection of plant, animal, or other materials from a natural setting:<br>• Field permit number<br>• Name of the institution or relevant body that granted permission	
**Data Availability**<br><br>Authors are required to make all data underlying the findings described fully available, without restriction, and from the time of publication. PLOS allows rare exceptions to address legal and ethical concerns. See the PLOS Data Policy and FAQ for detailed information.	No - some restrictions will apply

A Data Availability Statement describing where the data can be found is required at submission. Your answers to this question constitute the Data Availability Statement and **will be published in the article**, if accepted.<br><br>**Important:** Stating 'data available on request from the author' is not sufficient. If your data are only available upon request, select 'No' for the first question and explain your exceptional situation in the text box.<br><br>Do the authors confirm that all data underlying the findings described in their manuscript are fully available without restriction?	
**Describe where the data may be found in full sentences. If you are copying our sample text, replace any instances of XXX with the appropriate details.**<br><br>• If the data are **held or will be held in a public repository**, include URLs, accession numbers or DOIs. If this information will only be available after acceptance, indicate this by ticking the box below. For example: *All XXX files are available from the XXX database (accession number(s) XXX, XXX.)*.<br>• If the data are all contained **within the manuscript and/or Supporting Information files**, enter the following: *All relevant data are within the manuscript and its Supporting Information files.*<br>• If neither of these applies but you are able to provide **details of access elsewhere**, with or without limitations, please do so. For example:<br><br>*Data cannot be shared publicly because of [XXX]. Data are available from the XXX Institutional Data Access / Ethics Committee (contact via XXX) for researchers who meet the criteria for access to confidential data.*<br><br>*The data underlying the results presented in the study are available from (include the name of the third party*	The Skyline Tracksite is located within the Dinosaur Provincial Park Palaeontological Preserve.Due to the sensitivity of the site full site coordinate data are on file at the Royal Tyrrell Museum of Palaeontology, Drumheller, Alberta.Site number L2467.<br><br>All other relevant data are within the manuscript and its Supporting Information files.

Powered by Editorial Manager® and ProduXion Manager® from Aries Systems Corporation

and contact information or URL).<br>• This text is appropriate if the data are owned by a third party and authors do not have permission to share the data.<br><br>* typeset	
Additional data availability information:	

Powered by Editorial Manager® and ProduXion Manager® from Aries Systems Corporation

Manuscript

Click here to access/download;Manuscript;A ceratopsid-dominated tracksite_MANUSCRIPT.docx

A ceratopsid-dominated tracksite from the Dinosaur Park Formation (Campanian) at Dinosaur Provincial Park, Alberta, Canada

Phil R. Bell[1¶], Brian J. Pickles[2¶*], Sarah C. Ashby[2], Issy E. Walker[2], Sally Hurst[3], Michael Rampe[4], Paul Durkin[5], Caleb M. Brown[5,6,7]

[1]Palaeoscience Research Centre, School of Environmental and Rural Science, University of New England, Armidale, NSW, Australia.
[2]School of Biological Sciences, University of Reading, Whiteknights, Reading, UK.
[3]School of Natural Sciences, Macquarie University, Macquarie Park, NSW, Australia.
[4]Faculty of Arts, Macquarie University, Macquarie Park, NSW, Australia.
[5]University of Manitoba, Department of Earth Sciences, Winnipeg, Manitoba, Canada.
[6]Royal Tyrrell Museum of Palaeontology, Drumheller, Alberta, Canada.
[7]University of Alberta, Department of Biological Sciences, Edmonton, Alberta, Canada.

*Corresponding author:
E-mail: b.j.pickles@reading.ac.uk (BJP)

[¶]These authors contributed equally to this work.

Abstract

The badlands of Dinosaur Provincial Park (Alberta, Canada) are renowned for the exceptional abundance and diversity of Campanian-aged vertebrate body fossils, especially dinosaurs. Due to the steep exposures and rapid erosion, dinosaur tracks and trackways are considered extremely rare but have been recorded from a small number of concretionary casts, which pertain to hadrosaurids and a single tyrannosaurid. Here, we document the first multitaxic dinosaur footprint assemblage from the Dinosaur Park Formation based on a new locality that contains multiple individual ceratopsids, two tyrannosaurids, a possible ankylosaurian, and a small theropod-like taxon. Ceratopsid tracks are globally rare but dominate the new tracksite, suggesting gregarious behaviour, which is also supported by their regular spacing and parallel arrangement. The possible ankylosaurian track is identified (in part) on account of having three distinct pedal digits, consistent with the pedal anatomy of several Dinosaur Park ankylosaurids (*Euoplocephalus*, *Dyoplosaurus*) but differentiating it from previously-reported ankylosaurian tracks (*Tetrapodosaurus* isp.). Importantly, the new tracks are the first natural moulds (concave epirelief) found in Dinosaur Provincial Park, which, due to the unique geomorphology of the area, can only be recognised in outcrops where there are prominent sediment displacement rims. The new search image outlined here has already resulted in several subsequent trackway discoveries, and has the potential to transform ichnological studies in the Dinosaur Park Formation and related formations where badlands prevail.

Introduction

Dinosaur Provincial Park in southern Alberta, Canada, is unquestionably one of the premier localities worldwide for understanding Late Cretaceous terrestrial ecosystems [1]. The Park, as it is known, encompasses rocks of the Belly River Group (BRG) including the Oldman and Dinosaur Park formations, which have yielded hundreds of associated and articulated skeletons and literally millions of isolated bones and teeth, making it a model system for understanding dinosaur evolution, behaviour, biostratigraphy, and palaeoecology [1]. Despite the remarkable abundance of skeletal elements, dinosaur footprints and trackways are surprisingly rare [2-5]. This rarity is unquestionably linked to the steep exposures and high rates of erosion (2–4 mm/year; [6]) typical of badlands geomorphology [7], [8] and the unusual occurrence of concretionary tracks—track casts preserved as siderite concretions that protrude above the fine-grained sediments in which the footprint was originally emplaced [5]. Concretionary tracks, which are so far unique to several Late Cretaceous terrestrial deposits in southern Alberta, differ substantially in appearance from typical natural track moulds and casts, which occur as depressions or embossments, respectively, on bedding surfaces. Although they are probably more common than is typically recognised, the tendency of concretionary tracks to rapidly fall apart when exposed is likely linked to the apparent paucity of tracks in the Dinosaur Park Formation [5].

In 2024, one of us (PRB) discovered a new tracksite, the “Skyline Tracksite” comprising ‘typical’ natural mould (concave epirelief) tracks, which had heretofore not been identified in the Park. The first track was recognised based on an arc-shaped sediment displacement rim that protruded above the surface; excavation of the displacement rim revealed the first ceratopsid track (C1.1) and the adjacent track of a large theropod (T1.2) (Fig 1A). Further excavation exposed at least 20 partial and complete tracks including multiple trackways of ceratopsid and tyrannosaurid dinosaurs (Fig 1B, Fig 2).

Fig 1. Views of the Skyline Tracksite (TMP L2467) shortly after discovery (A) and following excavation (B). A) The sediment displacement rim (white arrows) of what turned out to be the first ceratopsid track (C1.1) was all that was visible when the site was first discovered. The inner part of the track was already partly exposed at the time the photo was taken. A tyrannosaurid track (black arrow, T1.2) had also been exposed in the foreground. B) View, looking southeast of the Skyline Tracksite following two days of excavation, note C1.1 and T1.2 indicated by white and black arrow respectively.

Fig 2. Schematic line drawing of Skyline Tracksite showing relative position and morphology of the mapped traces as of 2024. A) All mapped traces. B) traces likely pertaining to ornithischian dinosaurs. C) Traces likely pertaining to theropod dinosaurs. Dashed lines in B indicate possible track series and left/right pairs. Grid scale = 1 m.

Although several tracks and trackways have been previously reported from the Park, nearly all pertain to the concretionary tracks of hadrosaurids (a single tyrannosaurid track has also been reported [3]), which are the most abundant group of dinosaurs in the Park based on isolated skulls and partial or complete skeletons [9]. This new site represents the first multitaxic tracksite from the Park and includes the first ceratopsid, ankylosaurid, and small theropod tracks. Here, we describe the tracks and discuss the significance of this new site as it relates to the relative abundance of these track makers, possible gregarious behaviour, and track preservation at Dinosaur Provincial Park.

Materials and methods

All necessary permits were obtained for the described study, which complied with all relevant regulations (see Acknowledgements below).

Excavation

The Skyline Tracksite (TMP L2467) is located within the Dinosaur Provincial Park Palaeontological Preserve and is characterized by a resistant iron-cemented horizon overlain by a thick, highly friable mudstone (Fig 1). The site was excavated by hand using picks, awls, trowels, and hammers, to break up the friable mudstone, with the exposed surface cleaned using corn (i.e., whisk) brooms, paintbrushes and dustpans. Rubble was transported to below the track site in an attempt to stabilize the erosional edge of the track horizon and increase the longevity of the site. In some places, the track horizon was capped by a thin (0.5-1 cm) cemented layer, which could be removed in large sheets. In the 2024 field season, ~29 m^2 of the main track horizon was exposed, with the unexcavated back wall (15 m long) of the trackway continuing to the south, southeast and southwest. A much smaller excavation (~3 m^2) of what is likely the same track horizon took place 24 m to the southeast (125° from baseline), and confirms the continuation of the track site into the unexcavated hill to the south and southeast of the main site. Full site coordinate data are on file at the Royal Tyrrell Museum of Palaeontology, Drumheller, Alberta.

Mapping

Individual tracks were assigned an identifying prefix (A = ankylosaurid; C = ceratopsid; T = tyrannosaurid; U = unknown/unidentified trace) along with a number representing the individual trackmaker (1, 2, etc) and a final sequential number indicating the

position of the track in a trackway (if present). For example, the track number C1.2 identifies the second track in a sequence, or trackway, made by ceratopsid individual #1, whereas T2.1 identifies the first track made by tyrannosaurid individual #2.

The site was mapped using three different methods. Hand-drawn maps were created by first erecting a baseline, which was used to divide the surface into a grid with portable 1x1 m grid square (quadrat) with 10 cm subdivisions, largely following Rogers [10]. A chalk line was used to mark the baseline and grid boundaries on the trackway surface. Relevant features such as tracks, the erosional edge of horizon, and extent of the excavation were then drawn onto gridded paper, using the grid square and a plumb bob as a guide. Chalk was used to outline individual tracks to assist in mapping. Hand maps were drawn at two scales (20 cm: 1 m for individual 1x1 m grids, and 1 cm: 1 m for the overview map). The hand-drawn maps were then digitised, scaled, and combined into a single comprehensive map of the locality using Adobe Illustrator (V 23.0.6), which is presented in Fig 2.

The traditional grid square map was augmented using two digital mapping technologies: photogrammetry and differential GPS. Photogrammetric models were made by first photographing the entire track surface with a digital SLR camera, SX10 Mirrorless (Fujifilm: Macquarie Park, NSW, Australia) in an overlapping grid. An initial loop of the site was made, with the camera pointing towards the trackway site. Photos were taken at a constant walking pace, at a similar height and angle on each loop (where possible with varying terrain/elevation), ensuring that each photograph overlapped by at least a third. Additional loops and grid walks were conducted to ensure trackways were photographed from multiple angles and heights, and at a closer range to pick up additional details. Individual tracks were also photographed at a closer range.

A second round of photographs was taken following further excavation at the site a few weeks later using a digital SLR camera, X-S1 (Fujifilm: Bedford, UK) in an overlapping

grid. Photos were taken one pace apart, walking the same route four times (forwards and backwards with the camera facing forwards, then forwards and backwards with the camera pointed in the opposite direction), with the camera held at approximately 1.3 m high and pointed downwards at a 45° angle. This process was repeated multiple times.

Overlapping photos (camera settings: SX10 Mirrorless 18 mm lens, X-S1 24 mm lens) were then combined in Agisoft Metashape (version 2.1.3) to digitally reconstruct the tracksite in three-dimensions using standard photogrammetry techniques. These models, both of the entire tracksite, and individual tracks, were uploaded onto the Macquarie University Pedestal 3D gallery (**S1 Models:** https://mq.pedestal3d.com/r/bePfWqNGay/). To further enhance features of 3D photogrammetric models from Metashape, individual track models were loaded into MeshLab (2023.12) and rendered with a shading filter (‘toon.gdp’). In order to allow for the photogrammetry model to be georeferenced and to determine scale and orientation, a series of nine spatial landmarks (large rocks marked with flagging tape) were placed around the tracksite in places that would be captured in the models, but would not obscure trackway features. These marker points were then spatially located using a centimetre-scale differential GPS (Trimble Geo7X, TerraSync). Higher resolution photogrammetric models of individual representative tracks were also captured using a similar method but at a smaller scale.

In addition to the photogrammetric model, relevant features such as baseline posts, baselines, tracks, erosional edge of horizon, and extent of excavation were also traced using a centimetre scale differential GPS (Trimble Geo7X, TerraSync), rough[illegible]ollowing the methods of Brown and colleagues [11]. Linear features such as erosional and excavation edges, and partially uncovered tracks were mapped as open lines, while fully exposed tracks were mapped as polygons. Landmark (i.e., vertex) spacing for features ranges from 20-30 cm for erosional and excavation edges, to ~10 cm for tracks. Coordinate data were postprocessed

(Trimble Pathfinder) using the Cansel base station in Brooks, Alberta (35 km away), with all points having a postprocessed accuracy of <5 cm.

Measurements

Individual track features were measured in the field. Standard track measurements largely follow Therrien and colleagues [12] and Salisbury and colleagues [13] and included (where possible): compass bearing, track length, track width, free digit lengths, widths of digit bases, heel to digit tip lengths, heel to hypex lengths, divarication angles, length of digit III extension beyond the tips of II and IV (Fig 3).

Fig 3. Diagram showing morphometric measurements taken for each vertebrate trace. Measurements largely follow Therrien and colleagues [12] and Salisbury and colleagues [13]. Image modified from Therrien et al [12].

For trackway sequences—defined as two or more sequential tracks produced by the same individual—additional measurements included (where possible) pace and stride lengths, pace angulation, relative foot rotation, and compass bearing of the trackway midline. Most linear measurements (e.g., track length, track width, stride length) were taken on site using a flexible tape measure to the nearest mm, while orientations were taken using a Brunton compass to the nearest degree. Angular (i.e. digit divarication angles) and additional linear measurements were made using scaled photographs in ImageJ v.1.53.

Geological Setting and site description

Skyline Tracksite (TMP L2467)

The Belly River Group comprises, from oldest to youngest, the Foremost, Oldman, and Dinosaur Park formations [14] that form an eastward thinning clastic wedge of sediment shed from the Cordilleran to the west during an overall transgression of the Western Interior Seaway [15]. At the Park, the Dinosaur Park Formation is approximately 80 m thick, disconformably overlies the Oldman Formation, and is gradationally overlain by marine shales of the Bearpaw Formation (Fig 3A). Recent U-Pb zircon geochronology of bentonites (i.e. weathered volcanic ash) has constrained the age of the Dinosaur Park Formation as 76.47 +1.40/-0.084 to 74.44 +0.30/-0.11 Ma [16]. The Plateau Tuff (75.64 +/- 0.025 Ma) is a regionally extensive bentonite in the middle of the DPF that can be used as stratigraphic marker [16].

The Dinosaur Park Formation at the Park is characterized by fluvial channel-belt, floodplain, and coal deposits that record sedimentation on an alluvial plain westward of the contemporaneous Western Interior Seaway [17]. Paleochannel reconstructions reveal meandering rivers up to 10 m deep and approximately 200 m wide that constructed single-story channel-belt deposits 10 m in thickness and a few kilometers in width [18], [19]. Associated floodplain deposits are dominantly mudstones with thin sandstones and coals that record overbank sedimentation and paleosol development [20].

The Skyline Tracksite (TMP L2467) is located at 12U UTM E 0465298, N 5621098 in the upper portion of the Dinosaur Park Formation, stratigraphically above the Plateau Tuff (i.e. younger than 75.64 Ma) (Fig 4A). The elevation of the site is 704.88 m MSL (684.22 m HAE) and ~ 36 m above the contact with the Oldman Formation within the megaherbivore assemblage zone (MAZ-2a) of Mallon and colleagues [21] (partly equivalent to the *Lambeosaurus*-*Styracosaurus* dinosaur assemblage zone of Ryan and Evans [22] (Fig 4A).

The tracksite is located within a 12-m-thick succession of channel-belt deposits that overly older floodplain deposits (Fig 4B).

Fig 4. Geological context for Skyline Tracksite. A) Regional geological context for Skyline Tracksite (star) within the Dinosaur Park Formation and the *Prosaurolophus maximus-Styracosaurus albertensis* dinosaur megaherbivore faunal zone. Grey bar indicates the extent of the stratigraphic column in B. B) Stratigraphic section through Skyline Tracksite in the Dinosaur Park Formation. Trackway located at 8.4 m above base of section.

A 10-cm-thick mudstone hosts the trackway, which overlies a 2-m-thick succession of current-rippled sandstone and is overlain by 80-cm of interbedded fine current-rippled sandstone and bioturbated thin mudstone beds. These sediments record moderate-to-low energy deposition in an abandoned channel setting within a fluvial meandering channel-belt environment. The trackway host mudstone bed was likely formed as the result of overbank flooding and suspension settling of fine-grained sediment delivered to an abandoned channel environment. As water levels receded, the mud flat was subaerially exposed in the local topographic low of the abandoned channel. Subsequent moderate-to-low energy unidirectional flow through the abandoned channel environment led to burial and preservation of the trackway. Trace fossils in the overlying sediments are indicative of a quiescent, oxygenated environment consistent with a lacustrine setting contained in an abandoned channel, proximal to the active river channel and episodically flooded. The overall succession of channel-belt deposits exposed at the Skyline Tracksite are consistent with recent paleoenvironmental reconstructions from other study areas within the Park (e.g., [18], [19] that reveal a low-gradient meandering river and associated floodplain environment.

Following excavation, the track-bearing horizon measures 13 m long and up to 7 m wide, although the layer continues to the northwest and southeast of the present excavation into the adjacent slopes (Fig 1B; Fig 2A; Fig 5A).

Fig 5. Photogrammetrically generated Digital Elevation Model with heat map showing elevation of the Skyline Tracksite (A) and select individual prints (B-D). A) Model of excavated portion (as of 2024) of main trackway, with heatmap showing absolute elevation. Inset show location of B-D. B) Model of two adjacent traces, C1.1 (left) and T1.2 (right), with relative elevation heatmap. C) Model of A1.1, with relative elevation heatmap. C) Model of C4.1, with relative elevation. All models in plan view. Elevation legend in A, only pertains to A, as B-D are relative elevation only.

To the southwest (i.e., into the hill), the sedimentology of the track-bearing horizon grades from strongly indurated and iron-rich to more carbon-rich with abundant plant remains. As a result, the separation between the track-bearing surface and the overlying sandstone is less well developed making it difficult to follow (and excavate) in a southwesterly direction. Unlike the ironstone-rich (northeast) portion of the track-bearing horizon, which, except for the various ichnites, is smooth and featureless on its surface, the organic-rich southwesterly portion preserves common linear ripples (orientation = 102-282°). Unfortunately, current direction could not be measured from these ripples as they were consistently damaged during removal of the overlying beds due to the poor separation between layers. In addition, ichnites were less well defined in the organic-rich zone and had significantly less prominent sediment displacement rims. In contrast, tracks to the northeast show evidence of suction, including relatively deep tracks with steep-sided walls and prominent displacement rims. We interpret these changes in lithology and sedimentary

features to reflect a gradient in depositional environments from exposed, subaerial and muddy conditions (in the northeast) to shallow subaquatic but firmer conditions (in the southwest).

Although the purpose of this paper is to document the vertebrate ichnites found on the primary track-bearing horizon, common invertebrate traces (burrows) were found in the overlying mudstone within 30 cm of the track-bearing horizon. Three distinct invertebrate traces are preserved, all preserved as medium-grained sandstone infill with a host matrix. The most common traces are linear, vertical burrows (2-5 mm cross-sectional diameter) (Fig 6C), identified here as *Skolithos* [23]. Less common are larger unbranching, sub-horizontal, U-shaped burrows (5-9 mm diameter; 40-60 mm long) (Fig 6A-B – TMP 2024.012.0321), identified here as cf. *Rhizocorallium* [24].

Fig 6. Examples of the abundant invertebrate trace fossils found in the lower 30 cm of the mudstone overlying the vertebrate trace bearing horizon of the Skyline Tracksite. A-B) U-shaped marginal tubes of c.f. *Rhizocorallium*, both without (A) and with (B) *spreiten*. C) vertical burrows of *Skolithos.* Scale = 8 cm.

The majority of the U-shaped-burrows preserve only the infill; however, some preserve *spreiten*-like features between the marginal tubes. While the majority of *Rhizocorallium* occurrences are in marine settings, freshwater occurrences are known [25] and the u-shaped traces also bear broad similarity to sub-recent continental mayfly burrows [26]. Finally, the slightly higher portion of the mudstone with interfingering sandstone horizons, bears smaller, irregular, horizontal burrows (TMP 2024.012.0320), identified here as *Planolites*. Invertebrate traces were, however, not observed in the main track-bearing layer.

Results - Vertebrate ichnology

Ceratopsipes isp. – C1.1, C1.2, C2.1, C2.2, C3.1, C3.2, C4.1, C5.1, C5.2

Description

A total of nine tracks are assigned to this morphotype and at least eight represent sequential tracks made by four individuals (Fig 2B). Therefore, the tracks represent at least five individuals. All tracks pertain to the pedes; no definitive manus tracks were identified anywhere on the bedding surface. The best-preserved track (C1.1, a right pes) (Fig 5B; Figs 7A-B) is roughly circular in overall dimensions, slightly longer than wide with four broad, blunt digits and a prominent sediment displacement rim surrounding a broadly rounded but asymmetric (laterally skewed) ‘heel’ (= metatarsophalangeal pad) (Table 1).

Fig 7. Individual tracks of *Ceratopsipes* isp. A-B) trace C1.1 in photograph (A) and digital model (B). C) Photograph of trace C2.1. D) Photograph of trace C4.1. Scale = 8 cm. Roman numerals indicate interpreted digits.

Other tracks of this morphotype typically display a rounded ‘heel’ and have an overall circular shape but with various ill-defined digit impressions, often with shallow, trough-like toe drag marks (e.g., C1.1, C1.2, C2.1) making precise track length measurements difficult (Fig 7C). For example, C1.2, representing the left pes (and possibly the overprinted manus), is considerably longer than wide with a prominent posterior sediment displacement rim and poorly-defined digits I and II. The lateral digits (digits III and IV) are not discernible; instead, the area where they would be expected consists of a broad drag mark that extends from the anterolateral margin of the track.

Remarks

Broadly rounded pedal tracks with four blunt digits are characteristic of both ceratopsid and ankylosaurian trackmakers. Although manual proportions, especially in relation to the pes, are considered an important distinction between these two trackmakers [27], no conclusive manus impressions have yet been found at the Skyline Tracksite, TMP L2467. Therefore, the following discussion concerns only the well-preserved pedal tracks. Only a single ichnotaxon has been erected for both ceratopsid and ankylosaurian tracks: *Ceratopsipes goldenensis* and *Tetrapodosaurus borealis,* respectively [28], [27]. Based on the type material of both ichnotaxa, McCrea and colleagues ([27] considered *Ceratopsipes* tracks to be symmetrical with relatively short and proportionally broad digits. In contrast, *Tetrapodosaurus* has a less symmetrical pes, in part due to a short digit I compared to the other digits, although digits are in general proportionally longer, and more slender than in *Ceratopsipes*. Although the holotype pes of *Ceratopsipes* has a more attenuated 'heel' than the present examples from the Park, it is clear that natural variation—ranging from rounded to more V-shaped—exists in successive tracks from the same individual (including the trackway from which the holotype derives) and between other tracks assigned to *Ceratopsipes* [28-30]. The tracks from the Skyline Tracksite TMP L2467 share with *Ceratopsipes* a generally symmetrical pes with four relatively short, broad digits, therefore we assign the present tracks to *Ceratopsipes*, which is widely accepted as a ceratopsid track maker [28]. Absence of manual tracks precludes further specific diagnosis.

Ceratopsids are diverse and common components of the Dinosaur Park Formation, but with discrete biostratigraphic ranges within that formation [21]. TMP L2467 occurs within MAZ-2a, approx. 6 m above and 180 m in a straight line from Bonebed 42, a mixed assemblage dominated by the centrosaurine *Styracosaurus albertensis* [31], [32], [11],

Table 1. Skyline tracksite footprint data.

							Free digit length (cm)				Max digit width (cm)				Heel to digit tip (cm)				Heel to hypex (cm)			Divarication (°)			Digit extension (cm)		
**Taxon**	**Code**	**Side**	**Coordinates**	**Orientation (°)**	**Max length (cm)**	**Max width (cm)**	**I**	**II**	**III**	**IV**	**I**	**II**	**III**	**IV**	**I**	**II**	**III**	**IV**	**I-II**	**II-III**	**III-IV**	**I-II**	**II-III**	**III-IV**	**III beyond II and IV**	**Ichnofossil ID**	**Notes**
Ceratopsid	C1.1	R pes	D.8, 8.1	221	71	60	4†	8†	12†	11†	8†	9†	10†	10†	45†	55†	71	62†	44†	45†	47†	15†	17†	18†	11†	Ceratopsipes	
Ceratopsid	C1.2	?	F.3, 9.2	?	99	57	*	*	*	*	*	*	*	*	*	*	*	*	*	*	*	*	*	*	*		Morphology unclear; trackmaker may have slipped.
Ceratopsid	C2.1	L pes	E.9, 5.0	220	75	64	5.5†	10†	13†^	*	9†	11†	12†^	*	48†	66†	75	?	48†	55†	?	10†	19†	?	?	Ceratopsipes	
Ceratopsid	C2.2	R pes	G.1, 6.5	?	65	69	*	*	*	*	*	*	*	*	*	*	*	*	*	*	*	*	*	*	*		
Ankylosaur	A1.1	R pes	E.2, 3.7	205	67	61	na	13.5	19	6†	na	12	19	11†	na	55	67	55	na	45	52	na	24†	24†	18	Ankylosaurid	
Ankylosaur	A1.2	L pes	F.8, 3.8	?	?	?	*	*	*	*	*	*	*	*	*	*	*	*	*	*	*	*	*	*	*		Measurements not taken, still partially covered by overburden
Ceratopsid	C3.1	R pes	E.4, 2.4	203	73	74	4†	9†	14†	11†^	7†	9†	11†	9†^	52†	65†	73	62†	?	?	?	11†	17†	18†	14†	Ceratopsipes	Possible manus print that has been overprinted.
Ceratopsid	C3.2	L pes	F.9, 2.7	?	?	?	*	*	*	*	*	*	*	*	*	*	*	*	*	*	*	*	*	*	*		Measurements not taken, still partially covered by overburden
Ceratopsid	C4.1	L pes	E.5, 0.4	206	64	57*	5.5†	13†	14†^	*	10	11	10†^	*	54†	62†	64	*	45†	47.5†	?	15.5†	17†	?	?	Ceratopsipes	
Ceratopsid	C5.1	L pes ?	F.0, -1.2	?	74	67	*	*	*	*	*	*	*	*	*	*	*	*	*	*	*	*	*	*	*	Ceratopsipes	Measurements not taken, requires sediment removal
Ceratopsid	C5.2	R pes?	F.9, -1.5	?	?	?	*	*	*	*	*	*	*	*	*	*	*	*	*	*	*	*	*	*	*		Measurements not taken, still partially covered by overburden
Tyrannosaurid	T1.1	L pes	D.8, 4.4	300	45^	51^	na	?	32	18	na	?	13	?	na	37.5^†	45^	36†	na	18^†	16^†	na	?	46	21	Tyrannosaurid	
Tyrannosaurid	T1.2	R pes	D.2, 7.6	298	45	51	na	23	31	20	na	12	8	8	na	34†	45	33.5†	na	19.5†	13.5†	na	74	33	20	Tyrannosaurid	
Tyrannosaurid	T2.1	L pes	F.2, 7.4	290	?	?	na	?	32	25	na	?	15	13	na	?	?	?	na	?	?	na	?	31	?	Tyrannosaurid	
Small theropod-like	ST1	R pes?	E.8, 1.8	233	13	10	na	5	7.5	5	na	1	2	1.5	na	9†	13	8†	na	5†	5†	na	65	78	8.5†	Small theropod-like	Siding of DII vs DIV uncertain. Tentatively regarded as a right.

^ approximate due to imprecise preservation of one or more boundaries; * incomplete or not completely excavated; † estimated using imageJ

although *Chasmosaurus belli* [21], as well as potentially *Chasmosaurus russelli* and *Chasmosaurus irvinensis* [33], [34], also occur throughout MAZ-2a. Thus, there are at least two potential candidates for the *Ceratopsipes* trackmakers at TMP L2467, the centrosaurine *Styracosaurus albertensis* and a species of the chasmosaurine *Chasmosaurus*.

Ankylosauria – A1.1, A1.2 (Fig 2B, Fig 5D, Fig 8)

Description

A1.1 is a large tridactyl track tentatively identified as a right based on the reduced (digit IV) and its position relative to a second, partially-excavated track (A1.2) that is interpreted to be part of the same trackway (Fig 2B, Fig 5D, Fig 8).

Fig 8. Single track of possible ankylosaurid (A1.1). (A) photograph. (B) digital model. Scale = 10 cm. Roman numerals indicate interpreted digits.

A1.1 is well preserved, only slightly longer than wide (67 x 61 cm) and dominated by a large, round, symmetrical metatarsophalangeal pad measuring 45 cm in diameter and constituting ~72% of the total length of the track (Table 1; Fig 5D, Fig 8). Digit III, the largest of the digits, is blunt and rounded with a prominent proximal constriction. The remaining digits are less well demarcated; digit II is smaller than digit III, with a blunt terminus, a convex medial margin and a lateral medial margin. Owing to the constricted base of digit III, the hypex between digits III and II forms a broad, almost circular concavity in plan view. Digit IV is identifiable only as a weak, blunt prominence on the anteromedial margin of the track. There is no indication of the manus.

Remarks

The tridactyl form of A1.1 distinguishes it from ceratopsids and all known ankylosaurid tracks, which have a tetradactyl pes [28], [27]. Interestingly, the three blunt digits and symmetrical form resembles some stegosaurian tracks (*Deltapodus* isp., *Garbina* isp., *Stegopodus* isp.); however, there is no evidence of North American stegosaurs after the Jurassic [35]. *Deltapodus* also differs in having pedal tracks that are much more elongate than A1.1 [36]. Whereas tracks of *Stegopodus* are asymmetrical (as a result of posterior extension of a proximal pad at the base of digit IV) and have digits that barely project beyond the hypex [37], A.1.1 is notably more symmetrical with a rounded posterior track margin, and has digits that are comparatively elongate (especially digits III and ?IV). *Garbina*, originally named for Early Cretaceous thyreophoran tracks from Australia [13] shares with A1.1 large (up to 80 cm in *Garbina*), mesaxonic tracks with three blunt digits. However, A1.1 differs in having a digit III with a strong basal constriction, giving it a more rounded (rather than anteriorly tapering) outline. Hadrosaurids are ubiquitous components of the Dinosaur Park Formation fauna and their tracks have been reported previously [3], [5]. Although variations naturally exist due to variable substrate properties, kinematics, and preservation, Late Cretaceous hadrosaurid tracks (*Hadrosauropodus* isp.) have a tridactyl pes with blunt digits resembling A1.1; however, they tend to be wider than long with longer free digits that are ovoid, the long axes of which are oriented parallel to the track axis, and have rounded or bilobed heel impressions, with metatarsophalangeal pads that occupy, on average, 52% of the proportion of the total track length [38-40]. In contrast, A1.1 is longer than wide, with relatively small, short, rounded digits and a rounded metatarsophalangeal pad that occupies a greater portion (72%) of the total length of the track. Although ankylosaurians (including *Tetrapodosaurus* isp.) are normally associated with a tetradactyl pes, a number of Late Cretaceous ankylosaurids have a tridactyl pes, including *Euoplocephalus tutus*, *Dyoplosaurus acutosquameus* and *Pinacosaurus grangeri* [41-44]. Notably, both *Eupolocephalus* and

Dyoplosaurus come from the Dinosaur Park Formation: *Euoplocephalus* occurs throughout MAZ-2a, whereas *Dyoplosaurus* is known from only two occurrences in MAZ-1 [42]. A third taxon, the nodosaurid *Panoplosaurus mirus*, occurs throughout MAZ-1 [21]. Although the pes is unknown in *Panoplosaurus*, there is some indication that nodosaurids possessed tetradactyl pedes [43], which would potentially distinguish them from the tridactyl track A1.1. All previously-described ankylosaurian tracks have been attributed to the tetradactyl ichnotaxon *Tetrapodosaurus* [27], which is unsurprising given that the majority of these tracks come from 'middle' Cretaceous (Aptian–Turonian) strata [27], [4], [45]. By comparison, few Late Cretaceous ankylosaur tracks have been reported where tridactyl forms might conceivably exist [46], [47]. However, McCrea and colleagues [4] noted ankylosaurid tracks with tridactyl pedes in the Cenomanian Dunvegan Formation of British Columbia. These tracks were not described but were said to bear "some resemblance to Sternberg's [48] *Tetrapodosaurus* ichnosp." [4] and noted as having five manual digit impressions but only three pedal digit impressions [4]. A comparison between the only published image of these tracks [4], Fig 8) and A1.1 shows distinct similarities, especially in the overall rounded form of the pes with three blunt digit impressions and one digit (digit II?) that appears smaller and less developed than the remaining two.

Although tridactyl ankylosaur tracks have not previously been found, McCrea and colleagues [27] considered ankylosaur tracks to have digits that are longer and have less enclosing flesh than ceratopsid tracks, which appears to be the case in A1.1. Based on the above reasoning, we consider A1.1 a probable ankylosaurian, and most likely an ankylosaurid track, although in the absence of a more complete trackway, we refrain from a more specific diagnosis.

Tyrannosauridae T1.1, T1.2, T2.1 (Fig 2C, Fig 5B, Fig 9)

Description

Three large (~45 cm long) tridactyl theropod tracks were identified, two of which (T1.1 and T1.2) pertain to a partial trackway (Fig 2C, Fig 5B, Fig 9).

Fig 9. Individual tracks of tyrannosaurids. A-B) trace T1.2 in photograph (A) and digital model (B). C) Photograph of trace T1.1. D) Photograph of trace T2.1. Scale = 8 cm. Roman numerals indicate interpreted digits.

Based on the medial curvature of digit III, and a more posteriorly-situated hypex between digits III and IV compared to that of digits II and III [49], [39], T1.1 is identified as a left and T1.2 is a right. However, given that a significant portion of the track-bearing horizon is missing and the great distance (3.37 m) between the two tracks, at least two intervening tracks appear to be missing from the sequence (Fig 2C). Both T1.1 and T1.2 are tridactyl, mesaxonic with long, tapering digits that terminate in sharp ungual impressions (Fig 9). The claw trace of digit III in T1.1 is medially curved, whereas that of T1.2 is more symmetrical. There is no trace of a hallux. Digit II and III form a hypex that is close to 90° in T1.2, whereas digit II of T1.1 is only visible as shallow depression. The walls of the digits, especially digits III and IV, which are most deeply impressed, are steep-sided. The metatarsophalangeal impression is relatively small compared to overall track length (free digit length of digit III:track length = 0.7) The 'heel' of T1.2 tapers to an obtuse V-shaped angle, the apex of which is roughly in line with the long axis of digit III; the posterior border of T1.2 is less distinct and cannot be compared to T1.2. As best seen in T1.2, there is no visible medial notch at the base of digit II (an otherwise common theropod track feature), but this may be due to the poorly-imprinted posterior track margins of most tracks.

The third trace, T2.1, is moderately well preserved and represents the passage of a second individual; track orientations reveal both animals were travelling in a northwesterly direction (Table 1), in approximately parallel paths. Based on comparisons with T1.1–2, T2.1 is identified as a left pes. Digits III and IV are well defined but both the 'heel' and the entire digit II are poorly impressed and barely perceptible. Both digits III and IV are broad at their base and taper distally into sharp ungual impressions. Unlike in T2.1, the ungual of digit III does not curve medially but is more symmetrical.

Remarks

Potential trackmakers of the tridactyl theropod tracks include tyrannosaurids and possibly ornithomimids. Common ornithomimids in the Dinosaur Park Formation include *Struthiomimus* and *Ornithomimus*, although an indeterminate large-bodied ornithomimid, similar in proportions to the Mongolian taxon *Gallimimus*, has also been identified [50]. Based on the scaling of complete ornithomimid pedes, Enriquez and colleagues [51] determined the foot length of largest ornithomimids from the post-Cenomanian of Alberta (i.e., the large-bodied taxon described from the Dinosaur Park Formation by Longrich [50]) to be approximately 40 cm. Those authors therefore considered tridactyl theropod tracks ≥45 cm long to be too large to be produced by any known ornithomimid [51]. At ~45 cm in length, the large theropod tracks from TMP L2467 fall precisely on this cutoff, suggesting they too were made by tyrannosaurids. Tyrannosaurids from the Dinosaur Park Formation include the tyrannosaurine *Daspletosaurus* sp. and the more common albertosaurine *Gorgosaurus libratus* [52]. As their taxonomic distinction is based solely on osteological characters [53], [52], it is not possible to distinguish between them based only on tracks; we therefore consider the large theropod tracks at TMP L2467 to have been made by indeterminate tyrannosaurids.

There are currently two named ichnotaxa from the latest Cretaceous that unquestionably represent tyrannosaurid trackmakers: *Belloratipes fredlundi* and *Tyrannosauripus pillmorei* [54], [49]. McCrea and colleagues [49] diagnosed the tracks of *Belloratipes fredlundi* (in part) as mesaxonic, tridactyl, longer than wide, with broad digits that lack digital pads, and a large metatarsophalangeal pad compared to overall track length (free digit length of digit III:track length = 0.51; [49]). While most of these features apply to the theropod tracks from TMP L2467, the digits are proportionally longer and narrower with a small metatarsophalangeal pad (free digit length of digit III:track length = 0.7), which would argue against an assignment to *Belloratipes*. The tracks of *Tyrannosauripus* are similar to, but larger than *Bellotoripes* (40% longer, 20% wider), have faint digital pad impressions and a medially directed (as opposed to anteriorly directed) hallux [54], [49]. Thus, the theropod tracks from TMP L2467 cannot be attributed to *Tyrannosauripus.* In a broad sample of tyrannosaurid tracks from the Late Cretaceous of North America, Enriquez and colleagues [51] found that the free digit length of digit III is a function of absolute size: smaller tracks have a proportionately longer digit III than larger tracks. Given that the type series of *Belloratipes* represent larger tracks ($\mu\, TL = 60$ cm) than those described here ($\mu\, TL = 45$ cm)—*Tyrannosauripus* are larger still—, we would not expect them to share similar proportions. Instead, the TMP L2467 sample more closely resembles similar-sized ($TL = 40 - 49$ cm) unnamed tyrannosaurid tracks from the partly-coeval Wapiti Formation in central-western Alberta [51], [39] especially in having relatively long and narrow digits and a small metatarsophalangeal pad. We therefore refrain from assigning the present tracks to any existing ichnotaxon, preferring to leave them in open nomenclature

Small theropod-like taxon ST1 (Fig 10)

Description

ST1 is an isolated and shallowly-impressed tridactyl trace that was excavated in July 2024 (Fig 10), but because of the friable surface and low track relief had already eroded and was unrecognisable by August of the same year.

Fig 10. Single potential small theropod footprint (ST1) from the Skyline Tracksite. Scale = 5 cm.

As it was isolated, it is difficult to identify as either a right or left, although we treat it tentatively here as a right pes for the sake of the description. It is only slightly longer than wide (TL = 13 cm; TW = 10 cm), strongly mesaxonic, with long and tapering digits (Table 1). Divarication angles are relatively high (II^III = 65; III^IV = 78). Digit III is the longest digit and is straight, whereas digits II and IV are shorter, subequal in length, and curved along their long axis in a posterior direction; the curvature of digit II is stronger than that of digit IV. The metatarsophalangeal pad is moderately large (free digit length of digit III:track length $\mu = 0.58$), terminating in a broad V-shaped apex that is laterally offset with respect to the long axis of digit III. Digital pads are not discernible and there is no indication of a 'theropod' notch at the base of digit II.

Remarks

Relatively small tridactyl tracks with narrow digits are typically regarded as made by either small theropods or birds. A checklist of features for identifying avian traces given by Lockley and colleagues [55] includes: (1) resemblance to modern bird prints; (2) relatively small size (typically < 10 cm); (3) indefinite phalangeal pad impressions; (4) wide total digit divarication angle (II^IV = 110–120° or more); (5) a posteriorly-facing hallux; (6) thin claws;

(7) claws on digits II and IV curving away from digit III, and; (8) narrow digits relative to track length [55-57].

In contrast, tracks of non-avian theropods are regarded as having: (1) unequal lengths of digits II and IV, with digit IV being longer than digit II; (2) lower total digit divarication angle (II^IV < 90°); (3) a notch in the medial margin of the metatarsophalangeal pad close to the base of digit II, and: (4) medially curved unguals of digits II and III and a laterally curved ungual of digit IV [58]. Although footprint splay (FL/FW) has sometimes been considered, Xing and colleagues [57] found no evidence that this value differs between large avian and small (non-avian) theropod tracks. Because ST1 is an isolated and imperfectly-preserved trace, many of the above criteria are difficult to observe or to state with certainty (e.g., presence/curvature of unguals, presence of digital pads). Moreover, there is frequently considerable overlap in these features between non-avian theropods and birds either as a result of substrate consistency, biology or other features. For example, although total digit divarication of ST1 (II^IV = 143°) falls within the avian 'range', Xing and colleagues [57] found total divarication values of up to 120° in non-avian theropod tracks and cautioned against using this parameter as the sole distinguishing feature between possible trackmakers. Compounding this problem, Enriquez and colleagues [39] also considered thescelosaurids—which are known from the Dinosaur Park Formation [59] as capable of making small theropod-like tracks, although the tracks they described had lower divarication angles (II^IV = 57–87°) and comparatively broader digits. We also consider small-bodied pachycephalosaurids, which likely had functionally tridactyl pedes [60] and were a diverse and reasonably abundant component of the Dinosaur Park Formation fauna (e.g., [22], [61], as potential candidates. We therefore follow the conservative approach of Enriquez and colleagues [39] in referring ST1 to a small theropod-like trackmaker.

Discussion

Despite a rich history of exploration and unmatched abundance of body fossils, vertebrate ichnology in Dinosaur Provincial Park is still in its infancy [3]. Until relatively recently, only two isolated track casts had been collected (a hadrosaurid [TMP 1981.034.0001] and a tyrannosaurid [TMP 1993.036.0282]) although additional tracks and several badly eroded trackways were reported [2-4]. Following these earlier reports, Therrien and colleagues [5] documented two hadrosaurid trackways (including one first reported by McCrea [3]), highlighting the unusual preservation of concretionary tracks and forming a valuable new search image for footprints and trackways that has helped to recognise new trackways in other formations with similar geomorphology (e.g., Horseshoe Canyon Formation; [40]). Because of the steep exposures, 'popcorn' regolith, and rapid rates of erosion (2–4 mm/year; [6]) that typify the badlands of Dinosaur Provincial Park [6], [7], flat bedding surfaces are rarely exposed and, where present, localised depressions (such as dinosaur tracks) are rapidly filled by sediments. As a result, 'typical' tracks (natural moulds and casts) are unlikely to be recognised. Narrow ironstone benches, which are naturally resistant compared to the surrounding sandstones and mudstones, are common geomorphological features in the Park, yet localised depressions (including mouldic footprints) on these benches are invariably filled with sediments (either by the overlying track infill or from colluvial runoff), obscuring potential tracks. Tracks in the ironstone track-bearing layer at TMP L2467 were initially recognised only because the extrusive sediment rim of a single track was exposed above the overlying sandstone surface and only systematic excavation revealed the rest of the track. During the course of this study, however, we found that displacement rims could be readily identified on nearby ironstone ledges when they protruded above the overlying sandstone and that they could be easily excavated to reveal complete tracks. We therefore predict that 'typical' tracks, like those at the Skyline Tracksite,

are much more common within the area of DPP than previously assumed. Indeed, a brief exploration of the badlands near the Skyline Tracksite in August 2024, as well as ongoing fieldwork in the Park, have led to the discovery of several new tracksites by one of us (BJP) using this new search image (Fig 11).

Fig 11. Additional trackways found in the Dinosaur Park Formation subsequent to Skyline Tracksite discovery. A-C) 'Cathedral Tracksite' after initial discovery with minimal excavation (A) Sept 9, 2024, and following moderate excavation, Sept 12, 2024 (B), with track interpretation (C) showing a probable ceratopsian track (ce) and a large theropod track (t). Black arrows in A, B indicate the same sediment displacement rim for reference. B) Unexcavated isolated tridactyl print ('Mueller Tracksite' Aug 11, 2024, photo credit Andre Mueller). C) Unexcavated potential trackway ('Lambert Tracksite', Oct 25, 2024, photo credit Dawson Lambert).

Notably, new tracksites have now been found in both lower and upper parts of the Dinosaur Park Formation, and in varying local depositional environments, suggesting the occurrence of the Skyline Tracksite is not unique.

Once exposed, however, the thin ironstone layers preserving such tracks are prone to rapid erosion (similar to that observed on the concretionary ironstone tracks; [5]), likely influenced by the expansion and contraction of the bentonite-rich sediment layers during temperature extremes. Hence, once exposed at the surface, trackways such as the ones described here are unlikely to remain intact unless discovered and protected. Nevertheless, this new search image has the potential to dramatically alter the field of vertebrate palaeoichnology in the Park and for similar formations where badlands occur (e.g., Horseshoe Canyon and Milk River formations).

The discovery of the first concave epirelief tracks and trackways within Dinosaur Provincial Park reveals a snapshot of a Late Cretaceous multitaxic assemblage dominated by dinosaurs. We interpret the site as most likely representing an abandoned meandering channel, characterized by a lacustrine setting with episodic flooding. The environment would have provided the necessary conditions for footprint preservation and explains the presence of worm burrows typically associated with marine environments in the sedimentary layers above the tracksite. Although concretionary tracks attributed to hadrosaurs and a single tyrannosaurid track have previously been found, the tracksite reported here represents a more unusual assemblage including multiple individual ceratopsians, a possible ankylosaurian, at least two tyrannosaurids, and a small theropod-like taxon. Given the commonness of ceratopsids in uppermost Cretaceous strata of North America, it is surprising that relatively few of their tracks have been reported (e.g., [28], [46], [4], [30], [62]). Although it is impossible to tell the relative timing between the formation of the various tracks, several lines of evidence imply some level of synchronicity and, therefore, gregariousness or herding behaviour among *Ceratopsipes* trackmakers. In particular, the similar orientations, uniform spacing between individuals, and similar track characteristics (e.g., extrusive rims, slide marks) suggest the *Ceratopsipes* tracks were made at a time when sediment consistency was the same across much of the site, probably by a herd of animals traveling in the same direction. The *Ceratopsipes* tracks are furthermore oriented roughly perpendicular to and heading towards the ripple-laminated surface along the southwestern edge of the quarry, which indicates that these animals were moving towards the waterline, perhaps to drink. Taphonomic analyses of multiple extensive monodominant ceratopsid bonebeds that occur within the Park (including the nearby *Styracosaurus* bonebed [Bonebed 42]) provide strong evidence for gregariousness in this clade [63-66], specifically Centrosaurinae, a conclusion that is mirrored for ceratopsid bonebeds elsewhere across North America (e.g.,[67], [68],

[66][69]). However, it is acknowledged that such herding behaviour may only have occurred temporarily or at certain times of the year [66]. Nevertheless, it is not unexpected that the ceratopsids at the new tracksite may have been engaging in similar herding behaviour. If this truly does represent a ceratopsian herd, then *Styracosaurus albertensis* may be the more likely trace maker as there is abundant evidence for gregariousness in this taxon in the Dinosaur Park Formation, but not for *Chasmosaurus*.

Similarly, the tyrannosaurid tracks indicate two individuals walking adjacent to one another but parallel to the waterline (rather than towards it, as is the case for the ceratopsid tracks). Uniform orientations and similar track characteristics (e.g., extrusive sediment margins, especially at the hypices; poorly-impressed digit IV) again suggest the tracks were made relatively synchronously (but likely at a different time than the *Ceratopsipes* tracks). Possible gregariousness in North American tyrannosaurids has been interpreted previously from bonebed [70-72] and trackway evidence [49], [73], although, as with ceratopsids, the longevity of such gregarious associations may have varied [71]. While such an interpretation of the tyrannosaurid tracks at the Skyline Tracksite could be made, it is far from conclusive.

The absence of manus impressions from either the ceratopids or the presumed ankylosaurid is perplexing given that both are obligate quadrupeds. Trackways that favour the preservation of either the manus or pes (collectively referred to as either of manus-dominant [MDTs] or pes-dominant trackways [PDTs], respectively; [74]) are a well-known phenomenon among sauropods. Whereas MDTs are always the result of the pes failing to leave an impression, PDTs are more complex and may form either from the failure of the manus to leave an impression or as a result of overstepping by the hindfoot, whereby the manus print becomes partially or totally obscured [74]. Although, to our knowledge, neither MDTs nor PDTs have been reported for ankylosaurids or ceratopsids (see [37] and [13] for instances of PDTs among presumed stegosaurian trackmakers), similar reasons as those

posed for sauropod PDTs should also apply to these taxa. In their analyses of sauropod tracks, Falkingham and colleagues [74] proposed that animals with a posterior centre of mass (CM) would be more likely to produce PDTs. Although both ankylosaurians and ceratopsids had a CM that was placed well anterior to the acetabulum—as would be expected of a quadruped—it was closer to the hindlimb than to the forelimb, suggesting a greater distribution of weight on the pedes than on the mani [75], [76].

Finally, if our interpretation of the ankylosaurian track is correct, its similar alignment, spacing, orientation and preservation relative to the *Ceratopsipes* tracks could suggest the ceratopsians and ankylosaur were moving together in a single herd. In modern day ecosystems, mixed-species groups and aggregations are common and occur regularly in assemblages of fish, birds, large mammals, amphibians, and invertebrates [77]. Individual fitness can be enhanced through dilution benefits (reduced probability of being attacked by a predator; e.g. [78]) or detection benefits (increased probability of identifying a predator; e.g. [79]). Evidence is accumulating that access to social information [80], in this case from heterospecifics [81], appears to be a major driver of this phenomenon [82].

The most useful modern-day comparator for ornithischian macro-herbivores is probably large grazing mammals, and there are many studies on mixed-species assemblages on the African plains. A major explanation for the preference of some species for mixed-species groups appears to be as a defence strategy against shared large predators [83], [84], with individuals benefitting from the varied modes of sensory perception of heterospecifics, either by listening to their alarm calls [85] or by monitoring cues such as body posture [86]. This allows individuals to spend less time performing vigilance behaviours [87]. Interestingly, in seasonally wet and dry ecosystems, multi-species herding is reduced during the dry season and increases in the wet season, suggesting that this behaviour is influenced by resource (water) availability [88]. The tyrannosaur tracks within the Skyline Tracksite

indicate that large predators shared by ankylosaurs and ceratopsians are present, which corresponds to a key driver of multi-taxic herding behaviour in modern day large mammal herbivores (increased apex predator density; [84], [85], [87]).

Conclusions

The Dinosaur Park Formation in southern Alberta preserves one of the richest Late Cretaceous terrestrial communities in terms of the abundance and diversity of its vertebrate fossils. Yet despite more than a century of intensive collecting in the area of Dinosaur Provincial Park and the recovery of literally millions of vertebrate body fossils, fossil footprints and trackways have been elusive. While the steep, rapidly-eroding badlands geomorphology makes the Dinosaur Park Formation and related formations in the Western Interior of North America ideal for revealing body fossils, the absence of large planar surfaces (where tracks and trackways would be expected) has led to the widespread assumption that such ichnofossils are generally not preserved. Developing appropriate search images to find new fossil localities is an essential task for field-based palaeontology, and the recognition of peculiar concretionary tracks was an important development for prospecting in the badlands of southern Alberta. The new search image (protruding, often iron-rich sediment displacement rims) developed during the course of this study has already resulted in the discovery of several additional tracksites that are awaiting study. These preliminary findings indicate that vertebrate footprints are far more abundant in badland settings than has been previously accepted. The Skyline Tracksite described here underscores the importance of trackway data that can be gleaned from otherwise well explored formations, such as the Dinosaur Park Formation.

Acknowledgements

We thank Harris Ahmed, LJ Moss, Johana Simonova, and Jamie Stones (University of Reading) and Brady Holbach, Dawson Lambert, Urgon Snider, and Christopher West (RTMP) for assistance with site preparation. Dawson Lambert (RTMP) and Andre Mueller (Alberta Parks) found new tracksites following the discovery of TMP L2467. Fieldwork was conducted under Research and Collection Permit 24-409 (Alberta Parks) and Permit to Excavate Palaeontological Resources 24-047 (Alberta Culture). Jenifer Blacklaws and other Alberta Parks staff provided logistical support for fieldwork. We thank Nathan Enriquez for insightful discussions. Veronica Sew (Cansel) provided technical assistance with differential GPS correction. This work took place in the province that is known as Alberta. We gratefully recognize and honour Indigenous peoples' long-standing connections to these lands. This is the second paper arising from the International Palaeoecology Research Field Course led by BJP, CMB and PRB.

ORCID

Phil R. Bell https://orcid.org/0000-0001-5890-8183

Brian J. Pickles https://orcid.org/0000-0002-9809-6455

Sarah Ashby https://orcid.org/0009-0006-8966-8438

Issy E. Walker https://orcid.org/0009-0002-5289-2429

Sally Hurst https://orcid.org/0000-0002-1909-6685

Michael Rampe https://orcid.org/0000-0003-2855-2248

Caleb M. Brown https://orcid.org/0000-0001-6463-8677

References

1. Currie PJ, Koppelhus EB. Dinosaur Provincial Park: a spectacular ancient ecosystem revealed. Bloomington, IA, USA; Indiana University Press. 2005.
2. Sampson SD, Currie PJ. On the trail of Cretaceous dinosaurs. In: Ludvigsen R, editor. Life in stone: a natural history of British Columbia’s fossils. Vancouver, BC, Canada: UBC Press. 1996. pp 143-155.
3. McCrea RT, Currie PJ, Pemberton SG. Vertebrate ichnology. In: Currie PJ, Koppelhus EB, editors. Dinosaur Provincial Park: a spectacular ancient ecosystem revealed. Indiana Bloomington, IA, USA: University Press. 2005. pp 405–416.
4. McCrea RT, Buckley LG, Plint AG, Currie PJ, Haggart JW, Helm CW, et al. A review of vertebrate track-bearing formations from the Mesozoic and earliest Cenozoic of western Canada with a description of a new theropod ichnospecies and reassignment of an avian ichnogenus. New Mexico Museum of Natural History and Science Bulletin. 2014a;62: 5-93.
5. Therrien F, Zelenitsky DK, Quinney A, Tanaka K. Dinosaur trackways from the Upper Cretaceous Oldman and Dinosaur Park formations (Belly River Group) of southern Alberta, Canada, reveal novel ichnofossil preservation style. Can J Earth Sci. 2015;52(8): 630-641. doi: https://doi.org/10.1139/cjes-2014-0168
6. Campbell IA. Canadian landform examples — 3: Badlands of Dinosaur Provincial Park, Alberta. Can Geogr. 1987. 31(1):82-87. doi: https://doi.org/10.1111/j.1541-0064.1987.tb01592.x
7. Howard AD. Badlands. In: Abrahams AD, Parsons A, editors. Geomorphology of desert environments. Dordrecht, Netherlands: Springer. 1994. pp 213-242.

8. Gallart F, Solé A, Puigdefábregas J, Lázaro R. Badland systems in the Mediterranean. In: Bull JL, Kirkby MJ editors. Dryland Rivers: Hydrology and Geomorphology of Semi-arid Channels. Chichester, UK: John Wiley & Sons. 2002. pp 299-326.

9. Henderson DM, Tanke DH. Estimating past and future dinosaur skeletal abundances in Dinosaur Provincial Park, Alberta, Canada. Can J Earth Sci. 2010;47: 1291-1304. doi: **10.1139/E10-033**

10. Rogers RR. Collecting taphonomic data from vertebrate localities. In: Leiggi P, May P, editors. Vertebrate Paleontological Techniques. Cambridge, UK: Cambridge University Press. 1994. pp 47–58.

11. Brown CM, Herridge-Berry S, Chiba K, Vitkus A, Eberth DA. . High-resolution (centimetre-scale) GPS/GIS-based 3D mapping and spatial analysis of in situ fossils in two horned-dinosaur bonebeds in the Dinosaur Park Formation (Upper Cretaceous) at Dinosaur Provincial Park, Alberta, Canada. Can J Earth Sci. 2021;58: 225-246. doi: **10.1139/cjes-2019-0183**.

12. Therrien F, Zelenitsky DK, Tanaka K, Sloboda W.J. First hadrosaur trackway from the Upper Cretaceous (late Campanian) Oldman Formation, southeastern Alberta. In: Eberth DA, Evans DC, editors. Hadrosaurs. Bloomington, IA, USA: Indiana University Press. 2014. pp 532–539.

13. Salisbury SW, Romilio A, Herne MC, Tucker RT, Nair JP. . The dinosaurian ichnofauna of the Lower Cretaceous (Valanginian–Barremian) Broome Sandstone of the Walmadany area (James Price Point), Dampier Peninsula, Western Australia. J Vertebr Paleontol, 2016;36(sup1): 1-152. DOI: **https://doi.org/10.1080/02724634.2016.1269539**

14. Eberth DA, Hamblin AP. Tectonic, stratigraphic, and sedimentologic significance of a regional discontinuity in the upper Judith River Group (Belly River wedge) of

southern Alberta, Saskatchewan, and northern Montana. Can J Earth Sci. 1993;30: 174–200. doi: HTTPS://DOI.ORG/10.1139/E93-016

15. Eberth DA. The Geology. In: Currie PJ, Koppelhus EB, editors. Dinosaur Provincial Park: A Spectacular Ancient Ecosystem Revealed. Bloomington, IA, USA: Indiana University Press. 2005. pp 54–83.

16. Eberth DA, Evans DC, Ramezani J, Kamo SL, Brown CM, Currie PJ. et al. Calibrating geologic strata, dinosaurs, and other fossils at Dinosaur Provincial Park (Alberta, Canada) using a new CA-ID-TIMS U–Pb geochronology. Can J Earth Sci. 2023;60: 1627-1646. doi: HTTPS://DOI.ORG/10.1139/CJES-2023-0037.

17. Leckie DA, Smith DG. Regional setting, evolution, and depositional cycles of the Western Canada Foreland Basin. In: Macqueen RW, Leckie DA, editors. Foreland Basins and Fold Belts. Tulsa, OK, USA: American Association of Petroleum Geologists. 1992. Volume 55. doi: HTTPS://DOI.ORG/10.1306/M55563C2

18. Durkin PR, Hubbard SM, Holbrook J, Weleschuk Z, Nesbit P, Hugenholtz C, et al. Recognizing the product of concave-bank sedimentary processes in fluvial meander-belt strata. Sedimentology. 2020;67: 2819–2849. doi: HTTPS://DOI.ORG/10.1111/SED.12743

19. Mayo K, Silva RL, Durkin PR. Paleoenvironmental reconstruction of Late Cretaceous rivers, Dinosaur Park Formation, Alberta, Canada. Sediment Geol. 2023;457: 106499. doi: 10.1016/J.SEDGEO.2023.106499

20. Koster EH. Vertebrate taphonomy applied to the analysis of ancient fluvial systems. In*:* Ethridge FG, Flores RM, Harvey MD, editors. Recent Developments in Fluvial Sedimentology: Contributions from the Third International Fluvial Sedimentology Conference. Tulsa, OK, USA: The Society of Economic Palaeontologists and

Mineral Specialists, Special Publication. 1987;39: pp 1-9. doi: https://doi.org/10.2110/pec.87.39.0159

21. Mallon JC, Evans DC, Ryan MJ, Anderson JS. Megaherbivorous dinosaur turnover in the Dinosaur Park formation (upper Campanian) of Alberta, Canada. Palaeogeogr, Palaeoclimatol, Palaeoecol. 2012;350: 124-138. doi: 10.1016/j.palaeo.2012.06.024

22. Ryan MJ, Evans DC. Ornithischian dinosaurs. In: Currie PJ, Koppelhus EB, editors. *Dinosaur Provincial Park: A Spectacular Ancient Ecosystem Revealed*. Bloomington, IA, USA: Indiana University Press. 2005. pp 312–348.

23. Schlirf M. Upper Jurassic trace fossils from the Boulonnais (northern France). Geol Palaeontol. 2000;34: 145-213.

24. Knaust D. The ichnogenus Rhizocorallium: Classification, trace makers, palaeoenvironments and evolution. Earth Sci Rev. 2013;126: 1–47. doi: https://doi.org/10.1016/j.earscirev.2013.04.007

25. Fürsich FT, Mayr H. Non-marine Rhizocorallium (trace fossil) from the Upper Freshwater Molasse (upper Miocene) of southern Germany. Neues Jahrb Geol Palaontol Abh. Monatsh. 1981;6: 321-333. doi: 10.1127/njgpm/1981/1981/321

26. De C. Mangrove Ichnology of the Bay of Bengal Coast, Eastern India. Springer. Cham, Switzerland: International Publishing. 2019. doi: https://doi.org/10.1007/978-3-319-99232-7

27. McCrea RT, Lockley MG, Meyer CA. Global distribution of purported ankylosaur track occurrences. In: Carpenter K, editor. The Armored Dinosaurs. Bloomington, IA, USA: Indiana University Press. 2001. pp 413–454.

28. Lockley MG, Hunt AP. Ceratopsid tracks and associated ichnofauna from the Laramie Formation (Upper Cretaceous: Maastrichtian) of Colorado. J Vertebr Paleontol. 1995;15: 592-614. doi: https://doi.org/10.1080/02724634.1995.10011251

29. Lockley MG, Gierliński GD. Notes on a new ankylosaur track from the Dakota Group (Cretaceous) of Northern Colorado. In: Lockley MG, Lucas SG, editors. Fossil footprints of western North America. New Mexico Museum of Natural History and Science Bulletin. 2014;62: 301-306.

30. Lockley M, Gierliński G, Adach L, Schumacher B, Cart K. Newly discovered tetrapod ichnotaxa from the Upper Cretaceous Blackhawk Formation, Utah. New Mexico Museum of Natural History and Science Bulletin. 2018;79: 469-480.

31. Visser J. Sedimentology and taphonomy of a *Styracosaurus* bonebed in the Late Cretaceous Judith River Formation, Dinosaur Provincial Park, Alberta. D. Phil. Thesis, University of Calgary, Calgary, Alberta. 1986.

32. Ryan MJ, Holmes R, Russell AP. A revision of the late Campanian centrosaurine ceratopsid genus *Styracosaurus* from the Western Interior of North America. J Vertebr Paleontol. 2007;27(4): 944-962. doi: https://doi.org/10.1671/0272-4634(2007)27[944:AROTLC]2.0.CO;2

33. Campbell JA, Ryan MJ, Holmes RB, Schröder-Adams CJ. A Re-Evaluation of the Chasmosaurine Ceratopsid Genus *Chasmosaurus* (Dinosauria: Ornithischia) from the Upper Cretaceous (Campanian) Dinosaur Park Formation of Western Canada. PLoS One. 2016;11: e0145805. doi: https://doi.org/10.1371/journal.pone.0145805

34. Campbell JA, Ryan MJ, Schroder-Adams CJ, Holmes RB, Evans DC. Temporal range extension and evolution of the chasmosaurine ceratopsid '*Vagaceratops*' *irvinensis* (Dinosauria: Ornithischia) in the Upper Cretaceous (Campanian) Dinosaur Park Formation of Alberta. Vertebr Anat Morphol Palaeontol. 2019;7: 83-100. doi: dx.doi.org/10.18435/vamp29356

35. Maidment SC, Norman DB, Barrett PM, Upchurch P. Systematics and phylogeny of Stegosauria (Dinosauria: Ornithischia). J Syst Palaeontol. 2008;6: 367-407. doi: https://doi.org/10.1017/S1477201908002459

36. Castanera D, Mampel L, Cobos A. The complexity of tracking stegosaurs and their gregarious behavior. Sci Rep. 2024;14: 14833. doi: https://doi.org/10.1038/s41598-024-64298-9

37. Gierliński G, Sabath K. Stegosaurian footprints from the Morrison Formation of Utah and their implications for interpreting other ornithischian tracks. Oryctos. 2008;8: 29-46.

38. Lockley MG, Nadon G, Currie PJ. A diverse dinosaur-bird footprint assemblage from the Lance Formation, Upper Cretaceous, Eastern Wyoming: implications for ichnotaxonomy. Ichnos 2004;11: 229–249. doi: 10.1080/10420940490428625

39. Enriquez NJ, Campione NE, White MA, Fanti F, Sissons RL, Sullivan C, et al. The dinosaur tracks of tyrants aisle: An Upper Cretaceous ichnofauna from unit 4 of the Wapiti Formation (upper Campanian), Alberta, Canada. PLoS One. 2022;17: e0262824. doi: https://doi.org/10.1371/journal.pone.0262824

40. Powers MJ, Rhodes MM, Dyer AD, Mendonca SE, Wilkinson R, Hudgins MN, et al. The first hadrosaurid trackway from the Horseshoe Canyon Formation (Campanian/Maastrichtian) of Alberta, Canada. Ichnos 2023;30(3): 179-206. doi: https://doi.org/10.1080/10420940.2024.2307069

41. Arbour VM, Burns ME, Sissons RL. A redescription of the ankylosaurid dinosaur *Dyoplosaurus acutosquameus* Parks, 1924 (Ornithischia: Ankylosauria) and a revision of the genus. J Vertebr Paleontol. 2009;29: 1117-1135. doi: 10.1671/039.029.0405.

42. Arbour VM, Currie PJ. *Euoplocephalus tutus* and the diversity of ankylosaurid dinosaurs in the Late Cretaceous of Alberta, Canada, and Montana, USA. *PLoS One* 2013;8: e62421. doi: https://doi.org/10.1671/039.029.0405.

43. Currie PJ, Badamgarav D, Koppelhus EB, Sissons R, Vickaryous MK. Hands, feet, and behaviour in *Pinacosaurus* (Dinosauria: Ankylosauridae). Acta Palaeontol Pol. 2011;56: 489-504. doi: http://dx.doi.org/10.4202/app.2010.0055

44. Park JY, Lee YN, Currie PJ, Ryan MJ, Bell P, Sissons R, et al. A new ankylosaurid skeleton from the Upper Cretaceous Baruungoyot Formation of Mongolia: Its implications for ankylosaurid postcranial evolution. Sci Rep. 2021;11: 4101. doi: 10.1038/s41598-021-83568-4

45. Lockley MG, Kim KS, Lim JD. *Tetrapodosaurus* trackways from the Cretaceous of Colorado and Canada: observations on variability in ankylosaurian gaits. New Mexico Museum of Natural History and Science Bulletin. 2021;7: 227-235.

46. Fanti F, Bell PR, Sissons RL. A diverse, high-latitude ichnofauna from the Late Cretaceous Wapiti Formation, Alberta, Canada. Cretac Res. 2013;41: 256-269. doi: https://doi.org/10.1016/j.cretres.2012.12.010

47. Riguetti F, Citton P, Apesteguía S, Zacarías GG, Pereda-Suberbiola X. New ankylosaurian trackways (cf. *Tetrapodosaurus*) from an uppermost Cretaceous level of the El Molino Formation of Bolivia. Cretac Res. 2021;124: 104810. doi: https://doi.org/10.1016/j

48. Sternberg CM. Dinosaur tracks from Peace River, British Columbia. National Museum of Canada Annual Report, 1930. 1932: 59-85.

49. McCrea RT, Buckley LG, Farlow JO, Lockley MG, Currie PJ, Matthews NA, et al. A ‘terror of tyrannosaurs’: the first trackways of tyrannosaurids and evidence of

gregariousness and pathology in Tyrannosauridae. PLoS One. 2014b 9: e103613. doi: https://doi.org/10.1371/journal.pone.0103613

50. Longrich N. A new, large ornithomimid from the Cretaceous Dinosaur Park Formation of Alberta, Canada: implications for the study of dissociated dinosaur remains. Palaeontol. 2008;51: 983-997. doi: 10.1111/j.1475-4983.2008.00791.x

51. Enriquez NJ, Campione NE, Brougham T, Fanti F, White MA, Sissons RL, et al. Exploring possible ontogenetic trajectories in tyrannosaurids using tracks from the Wapiti Formation (upper Campanian) of Alberta, Canada. J Vertebr Paleontol. 2020;40: e1878201. doi: https://doi.org/10.1080/02724634.2021.1878201

52. Currie PJ. Theropods, including birds. In: Currie PJ, Koppelhus EB, editors. Dinosaur Provincial Park: a spectacular ancient ecosystem revealed. Bloomington, IA, USA: Indiana University Press. 2005 pp. 367–397.

53. Currie PJ. Cranial anatomy of tyrannosaurid dinosaurs from the Late Cretaceous of Alberta, Canada. Acta Palaeontol Pol. 2003;48: 191–226. doi: http://dx.doi.org/10.1017/CBO9780511608377.023

54. Lockley MG, Hunt AP. A track of the giant theropod dinosaur Tyrannosaurus from close to the Cretaceous/Tertiary Boundary, northern New Mexico. Ichnos 1994;3: 213–218.

55. Lockley MG, Yang SY, Matsukawa M, Fleming F, Lim SK. The track record of Mesozoic birds: evidence and implications. Philos Trans R Soc Lond B Biol Sci. 1992;336: 113-134. doi: https://doi.org/10.1098/rstb.1992.0051

56. Falk AR, Martin LD, Hasiotis ST. . A morphologic criterion to distinguish bird tracks. J Ornithol. 2011;152: 701-716. doi: https://doi.org/10.1007/s10336-011-0645-x

57. Xing L, Buckley, LG, McCrea RT, Lockley, MG, Zhang J, Piñuela L, et al. Reanalysis of *Wupus agilis* (Early Cretaceous) of Chongqing, China as a large avian trace:

differentiating between large bird and small non-avian theropod tracks. *PLoS One*, 2015;10(5): e0124039. doi: HTTPS://DOI.ORG/10.1371/JOURNAL.PONE.0124039

58. Wright JL. Bird-like features of dinosaur footprints. In: Currie PJ, Koppelhus EB, Shugar MA, Wright JL, editors. Feathered dragons: studies on the transition from dinosaurs to birds. Bloomington, IA, USA: Indiana University Press. 2004. pp 167–181.

59. Brown CM, Russell AP, Evans DC, Ryan, MJ. New data on the diversity and abundance of small-bodied ornithopods (Dinosauria: Ornithischia) from the Belly River Group (Campanian) of Alberta. J Vertebr Paleontol. 2013;33: 495-520. doi: 10.1080/02724634.2013.746229.

60. Maryanska T, Chapman RE, Weishampel DB. Pachycephalosauria. In: Weishampel DB, Dodson P, Osmólska H, editors. *The Dinosauria*. Berkeley, CA, USA: University of California Press. 2004. pp 464–477.

61. Woodruff DC, Schott RK, Evans DC. Two new species of small-bodied pachycephalosaurine (Dinosauria, Marginocephalia) from the uppermost Cretaceous of North America suggest hidden diversity in well-sampled formations. Pap Palaeontol. 2023;9(6): e1535. doi: HTTPS://DOI.ORG/10.1002/SPP2.1535

62. Stewart DG, Druckenmiller PS, Erickson GM, Benowitz JA, Capps DM, Knight CL, et al. Vertebrate ichnology and palaeoenvironmental associations of Alaska's largest known dinosaur tracksite in the Cretaceous Cantwell Formation (Maastrichtian) of Denali National Park and Preserve. Hist Biol, 2023;36(8): 1540-1556. doi: HTTPS://DOI.ORG/10.1080/08912963.2023.2221267

63. Currie PJ, Dodson P. Mass death of a herd of ceratopsian dinosaurs. In: Reif WE, Westphal F, editors. Third Symposium on Mesozoic Terrestrial Ecosystems. 1984. Tübingen, Germany; Attempto Verlag. pp 61–66.

64. Ryan M J, Russell AP, Eberth DA, Currie PJ. The taphonomy of a *Centrosaurus* (Ornithischia: Ceratopsidae) bone bed from the Dinosaur Park Formation (Upper Campanian), Alberta, Canada, with comments on cranial ontogeny. Palaios. 2001;16(5): 482-506. doi: https://doi.org/10.1016/j.cretres.2021.104810

65. Eberth DA, Getty MA. Ceratopsian bonebeds: occurrence, origins, and significance. In: Currie PJ, Koppelhus EB, editors. Dinosaur Provincial Park: a spectacular ancient ecosystem revealed. Bloomington, IA, USA: Indiana University Press. 2005. pp 501–536.

66. Hunt RK, Farke AA. Behavioral interpretations from ceratopsid bonebeds. In: Ryan MJ, Chinnery-Allgeier B, Eberth DA eds. New Perspectives on Horned Dinosaurs: The Royal Tyrrell Museum Ceratopsian Symposium. Bloomington, IA, USA: Indiana University Press. 2010. pp 447-455.

67. Currie PJ, Langston W, Tanke DH. A new horned dinosaur from an Upper Cretaceous bone bed in Alberta. Ottawa, Ontario, Canada: NRC Research Press. 2008. pp 144.

68. Mathews JC, Brusatte SL, Williams SA, Henderson MD. The first *Triceratops* bonebed and its implications for gregarious behavior. J Vertebr Paleontol. 2009;29: 286-290. doi: 10.1080/02724634.2009.10010382

69. Scott SH, Ryan MJ, Evans DC. Postcranial description of *Wendiceratops pinhornensis* and a taphonomic analysis of the oldest monodominant ceratopsid bonebed. Anat Rec. 2023;306(7): 1824-1841. DOI: https://doi.org/10.1002/ar.25045

70. Currie PJ. Possible evidence of gregarious behavior in tyrannosaurids. *Gaia.* 1998;15: 271–277. doi: https://doi.org/10.7939/R3348GX03

71. Currie PJ, Eberth DA. On gregarious behavior in *Albertosaurus*. Can J Earth Sci. 2010;47: 1277-1289. doi: https://doi.org/10.1139/E10-072

72. Titus AL, Knoll K, Sertich JJ, Yamamura D, Suarez CA, Glasspool IJ, et al. Geology and taphonomy of a unique tyrannosaurid bonebed from the upper Campanian Kaiparowits Formation of southern Utah: implications for tyrannosaurid gregariousness. PeerJ, 2021;9: e11013. doi: https://doi.org/10.7717/peerj.11013

73. Henderson DM, Borkovic B, Sanchez J, Kowalchuk AL. A busy time at the beach: multiple examples of gregarious dinosaur behaviour inferred from a set of trackways from the Late Cretaceous of Alberta, Canada. Can J Earth Sci. 2022;59: 608-622. doi: https://doi.org/10.1139/cjes-2021-0069

74. Falkingham PL, Bates KT, Mannion PD. Temporal and palaeoenvironmental distribution of manus-and pes-dominated sauropod trackways. J Geol Soc London. 2012;169: 365-370. doi: https://doi.org/10.1144/0016-76492011-019

75. Maidment SC, Henderson DM, Barrett PM. What drove reversions to quadrupedality in ornithischian dinosaurs? Testing hypotheses using centre of mass modelling. Sci Nat. 2014;101: 989-1001. doi: 10.1007/s00114-014-1239-2

76. Mallon JC, Henderson DM, McDonough CM, Loughry WJ. A “bloat-and-float” taphonomic model best explains the upside-down preservation of ankylosaurs. Palaeogeogr, Palaeoclimatol, Palaeoecol. 2018;497: 117-127. doi: https://doi.org/10.1016/j.palaeo.2018.02.010

77. Carlson NV, Freeberg TM, Goodale E, Theo AH. Mixed-species groups and aggregations: shaping ecological and behavioural patterns and processes. Philos Trans R Soc B Biol Sci. 2023;378: 20220093. doi: https://doi.org/10.1098/rstb.2022.0093

78. Hamilton WD. Geometry for the selfish herd. J Theor Biol. 1971;31: 295–311. doi: https://doi.org/10.1016/0022-5193(71)90189-5

79. Pulliam HR. On the advantages of flocking. J Theor Biol. 1973;38: 419 422. doi: 10.1016/0022-5193(73)90184-7

80. Dall SRX, Giraldeau LA, Olsson O, McNamara JM, Stephens DW. Information and its use by animals in evolutionary ecology. Trends Ecol Evol. 2005;20: 187-193. doi: https://doi.org/10.1016/j.tree.2005.01.010

81. Oliveira RF, Bshary R. Expanding the concept of social behavior to interspecific interactions. Ethol. 2021;127: 758–773. doi: https://doi.org/10.1111/eth.13194

82. Meise K, Franks DW, Bro-Jorgensen J. Alarm communication networks as a driver of community structure in African savannah herbivores. Ecol Lett. 2020;23: 293–304. doi: https://doi.org/10.1111/ele.13432

83. Magrath RD, Haff TM, Fallow PM, Radford AN. Eavesdropping on heterospecific alarm calls: from mechanisms to consequences. Biol Rev. 2015;90: 560-586. doi: 10.1111/brv.12122

84. Makin DF, Chamaillé-Jammes S, Shrader AM. Herbivores employ a suite of antipredator behaviours to minimize risk from ambush and cursorial predators. Anim Behav. 2017;127: 225-231. doi: https://doi.org/10.1016/j.anbehav.2017.03.024

85. Palmer MS, Gross A. Eavesdropping in an African large mammal community: antipredator responses vary according to signaller reliability. Anim Behav. 2018;137: 1-9. doi: https://doi.org/10.1016/j.anbehav.2017.12.018

86. Schmitt MH, Stears K, Shrader AM. Zebra reduce predation risk in mixed-species herds by eavesdropping on cues from giraffe. Behav Ecol. 2016;27(4): 1073–1077. doi: https://doi.org/10.1093/beheco/arw015

87. Stears K, Schmitt MH, Wilmers CC, Shrader AM. Mixed-species herding levels the landscape of fear. *Proc R Soc B.* 2020;287: 1922, 20192555. doi: https://doi.org/10.1098/rspb.2019.2555

88. Kiffner C, Kioko J, Leweri C, Krause S. Seasonal Patterns of Mixed Species Groups in Large East African Mammals. PLoS One. 2014;9: e113446. doi: https://doi.org/10.1371/journal.pone.0113446

Supporting Information

S1 Models. 3D Model Gallery. https://mq.pedestal3d.com/r/bePfWqNGay/

Fig 1

Click here to access/download;Figure;FIGURE_1_Excavation.tif

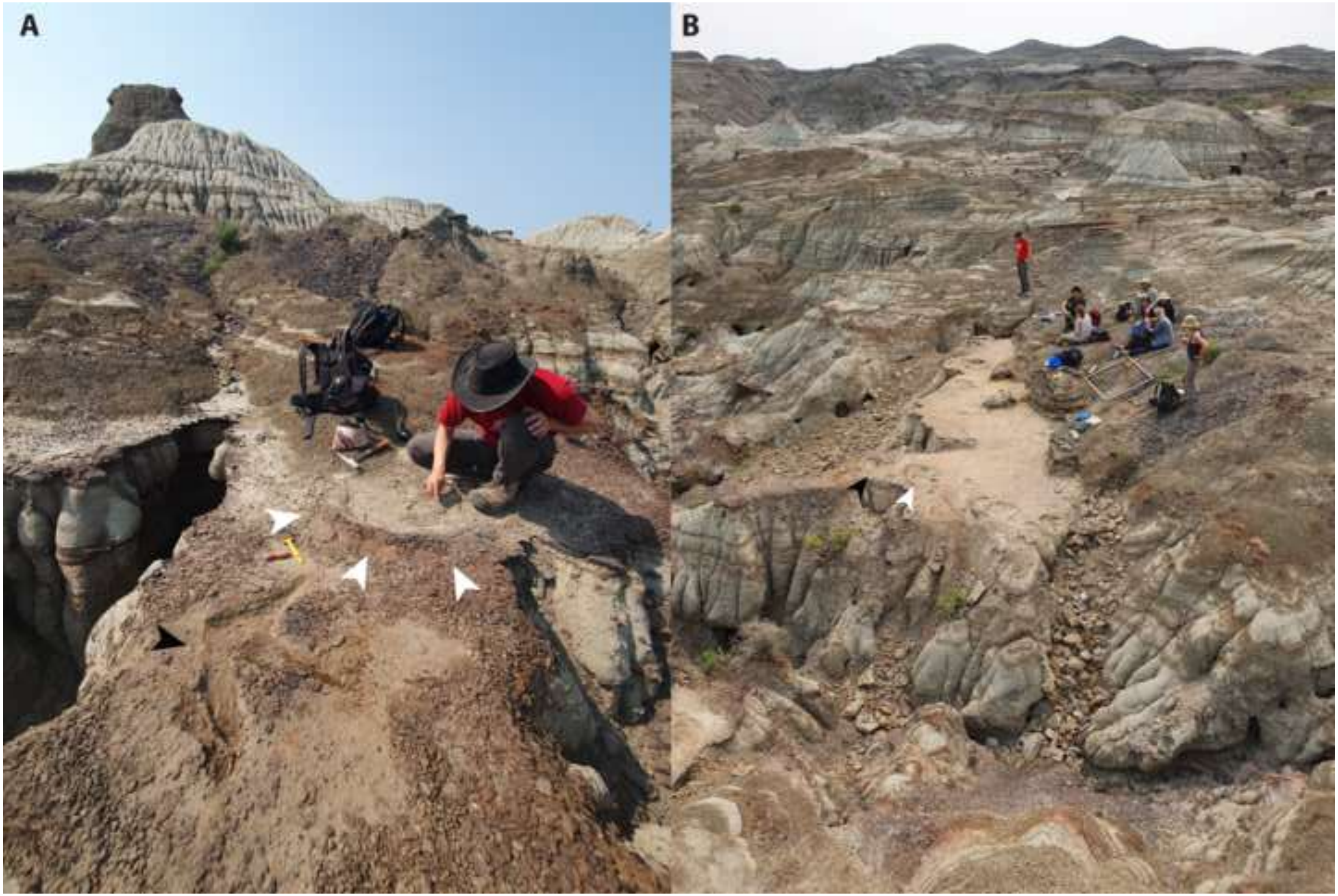

Fig 2

Click here to access/download;Figure;FIGURE_2_Composite_Map.tif

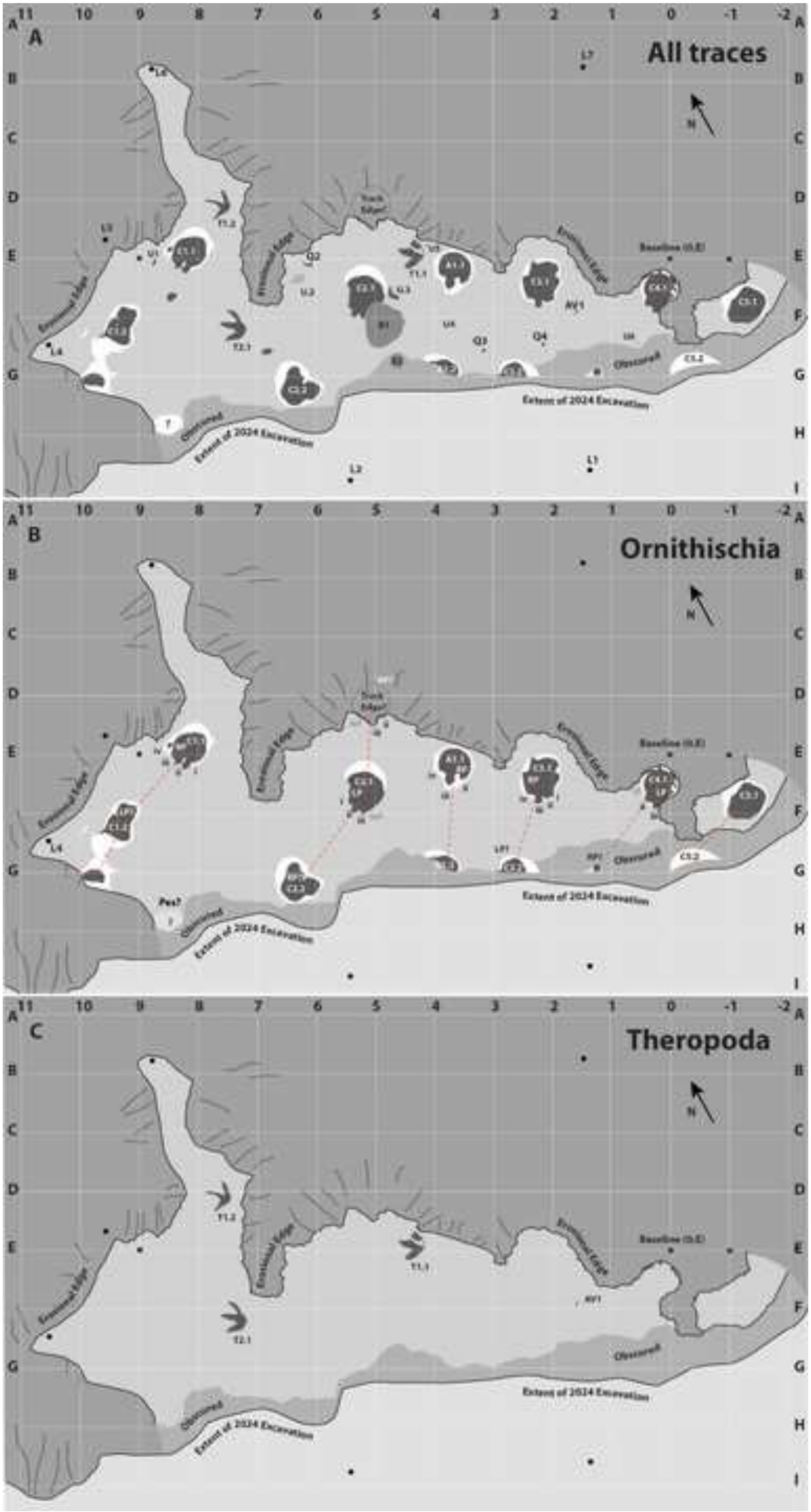

Fig 3

Click here to access/download;Figure;FIGURE_3_measurement.tif

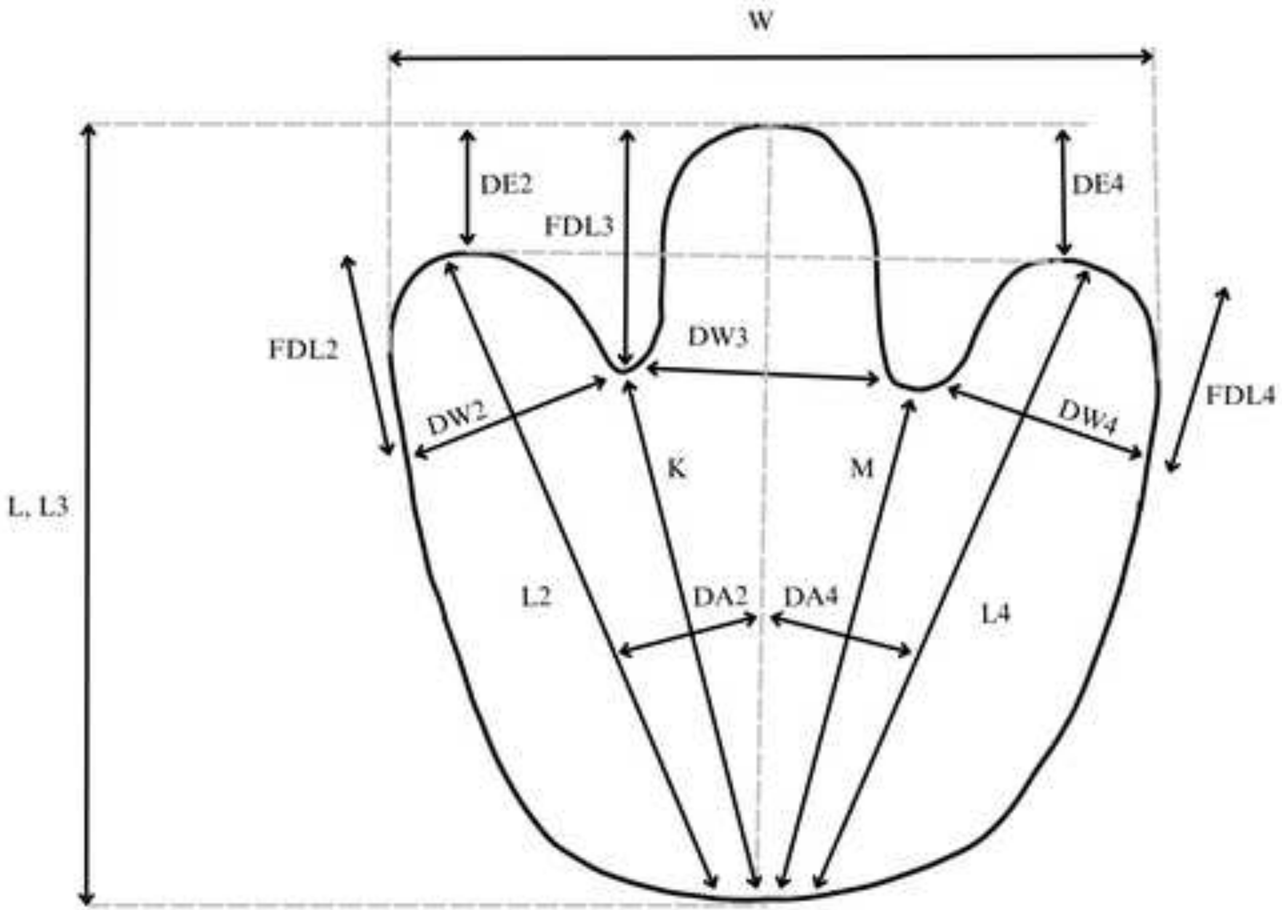

Fig 4

Click here to access/download;Figure;FIGURE_4_Geology.tif

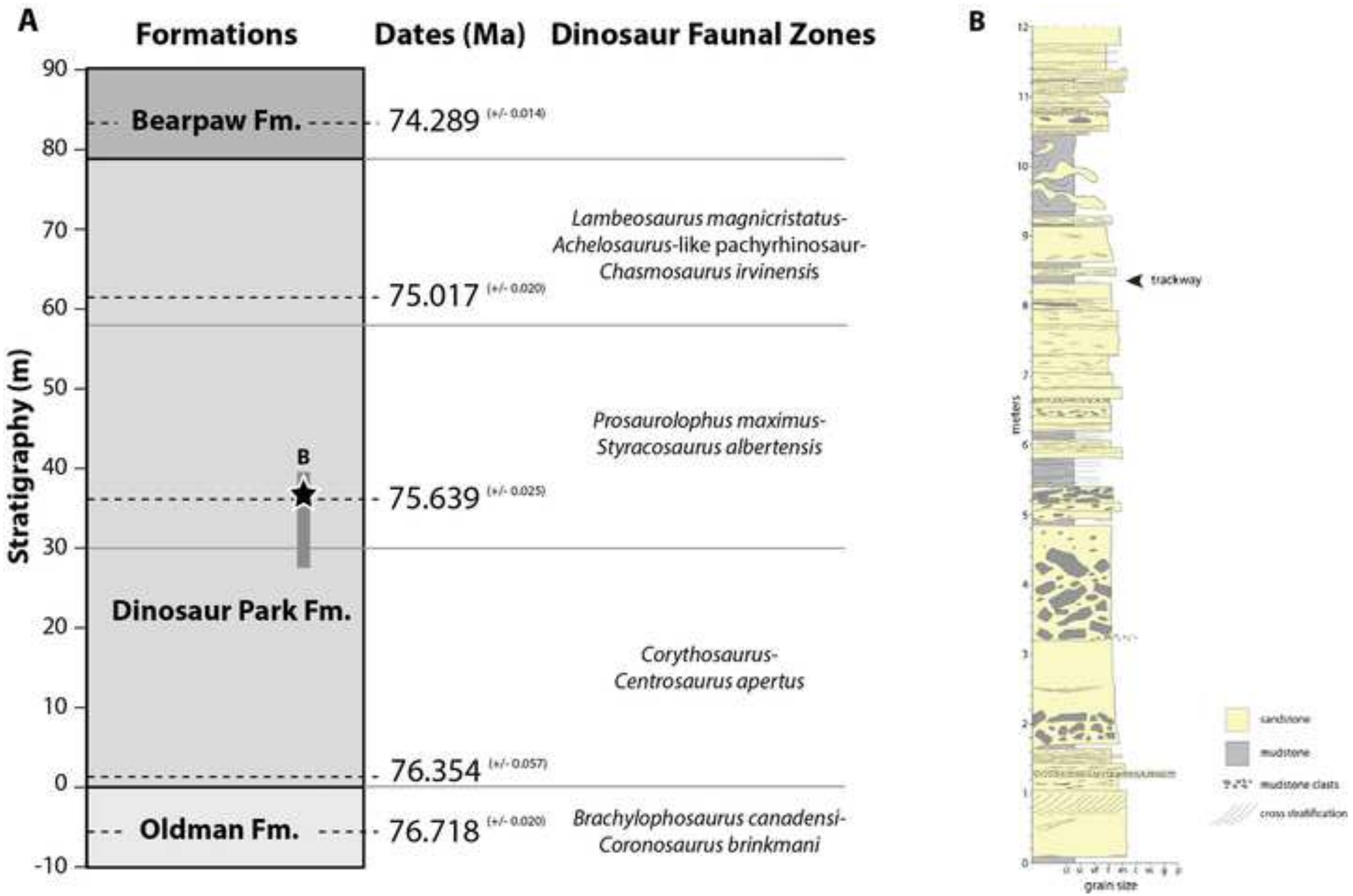

Click here to access/download;Figure;FIGURE_5_DEM.tif

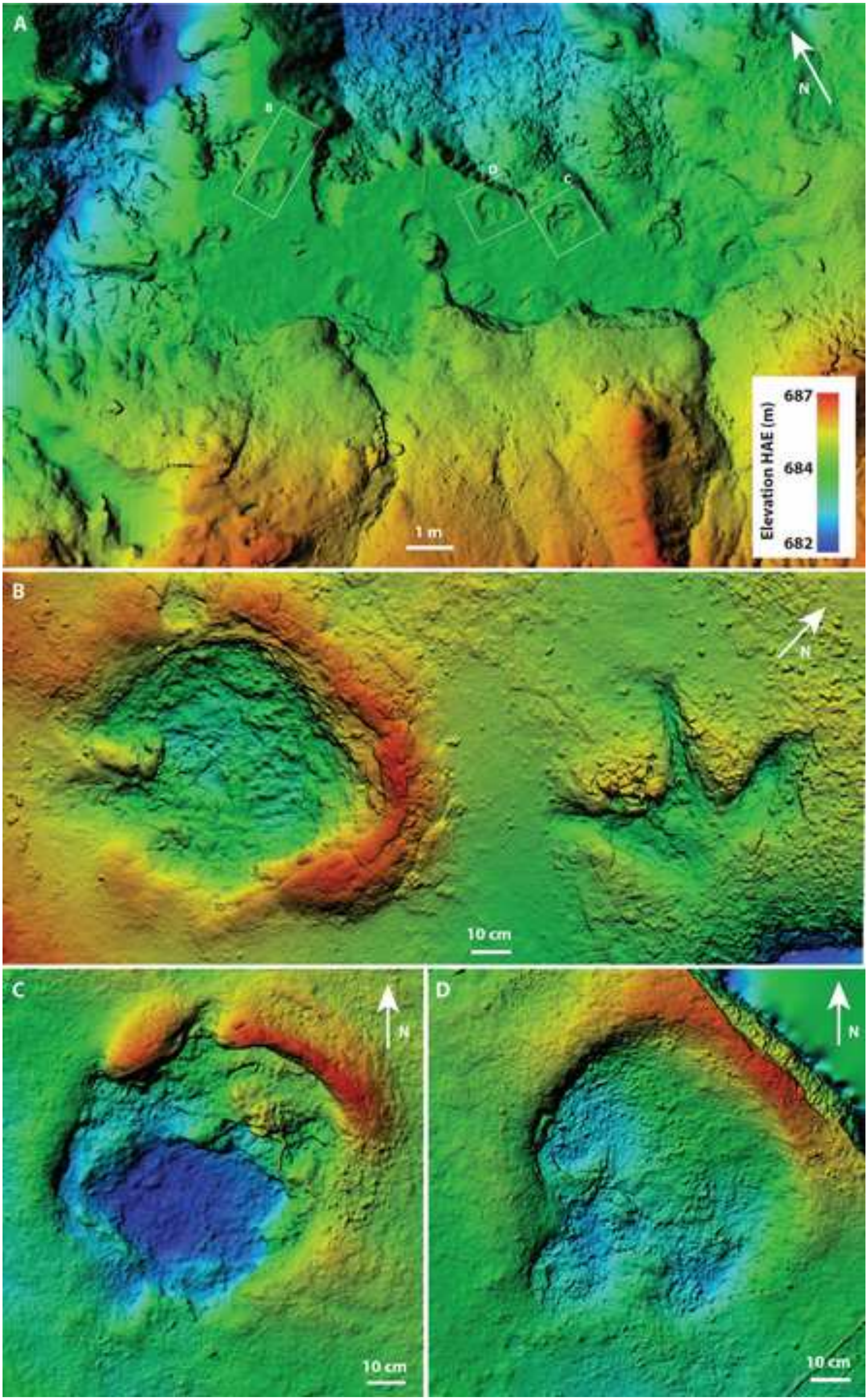

Fig 6

Click here to access/download;Figure;FIGURE_6_invert traces.tif

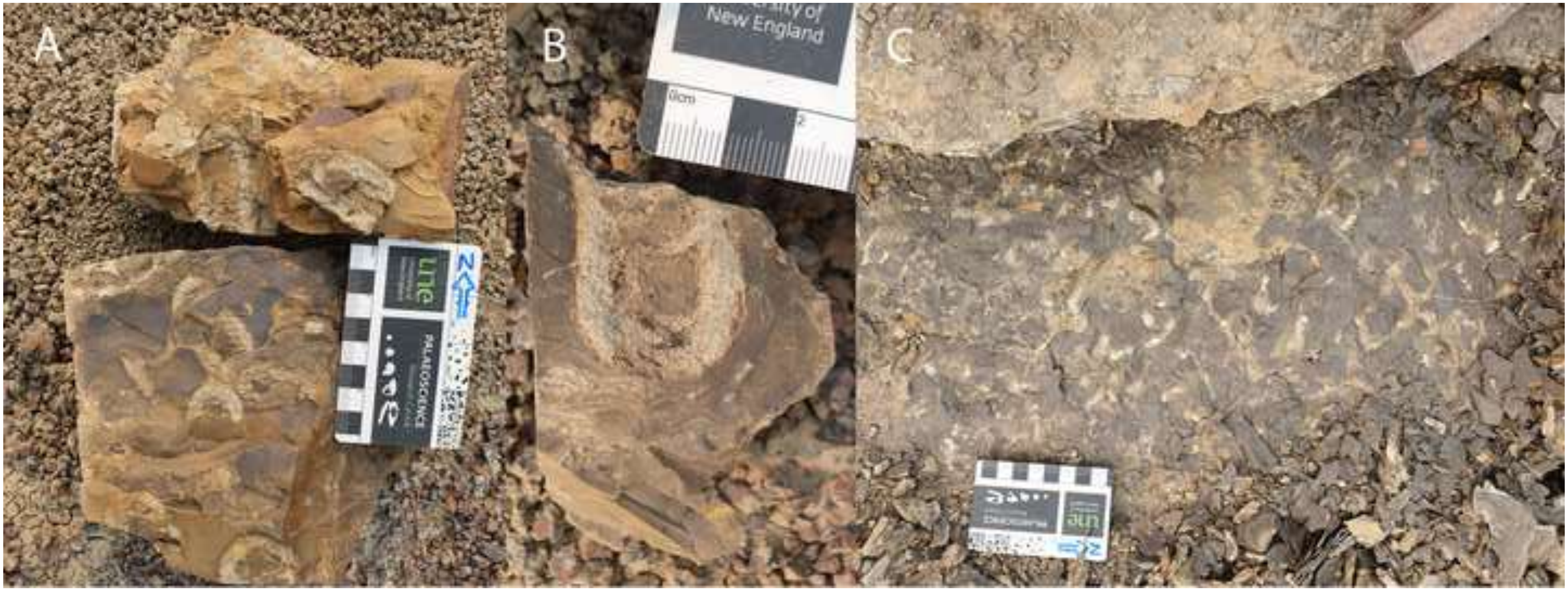

Fig 7

Click here to access/download;Figure;FIGURE_7_ceratopsid tracks.tif

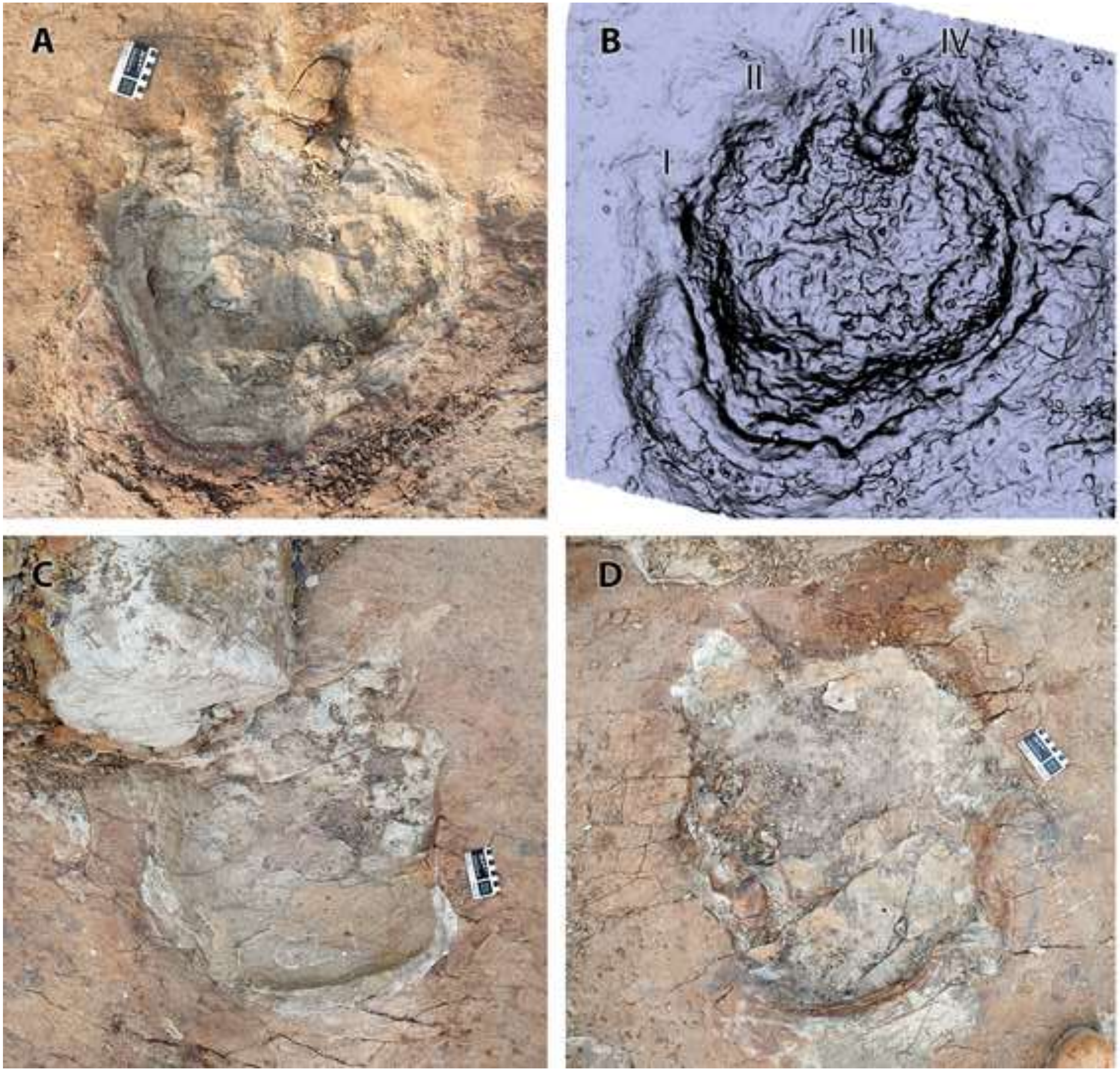

Click here to access/download;Figure;FIGURE_8_Ankylosaur tracks.tif

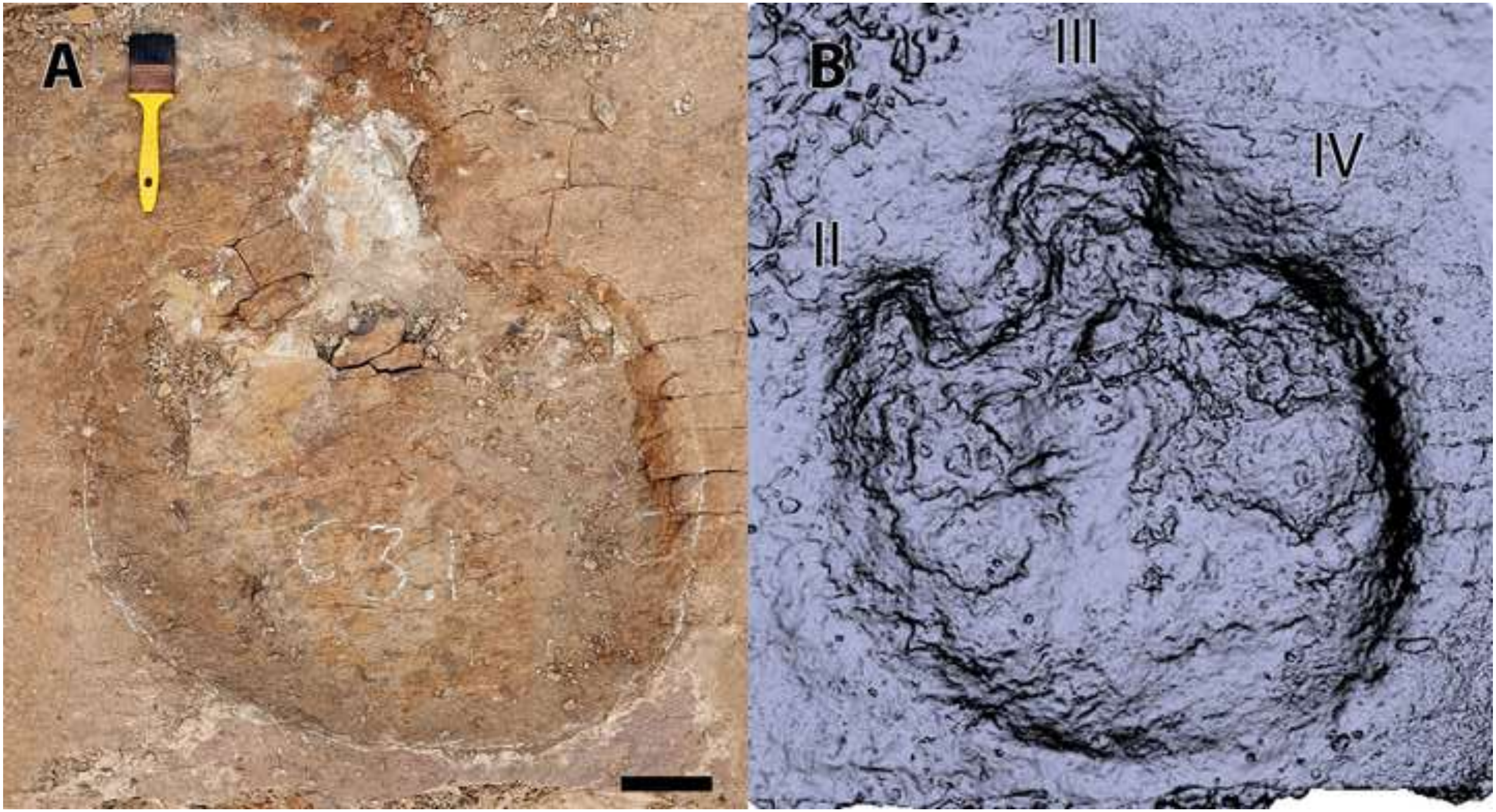

Fig 9

Click here to access/download;Figure;FIGURE_9_Tyrannosaur tracks.tif

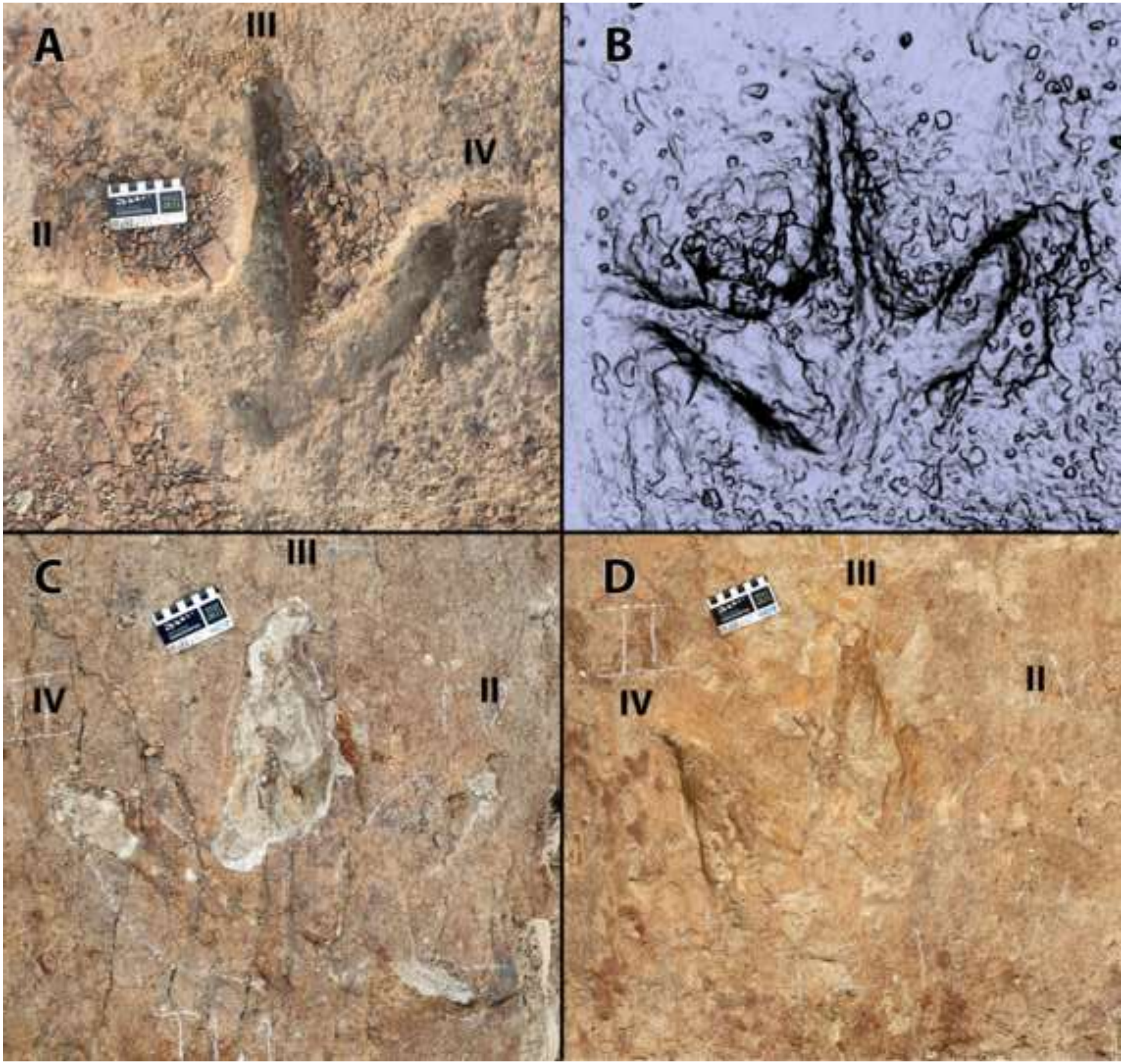

Click here to access/download;Figure;FIGURE_10 small theropod.tif

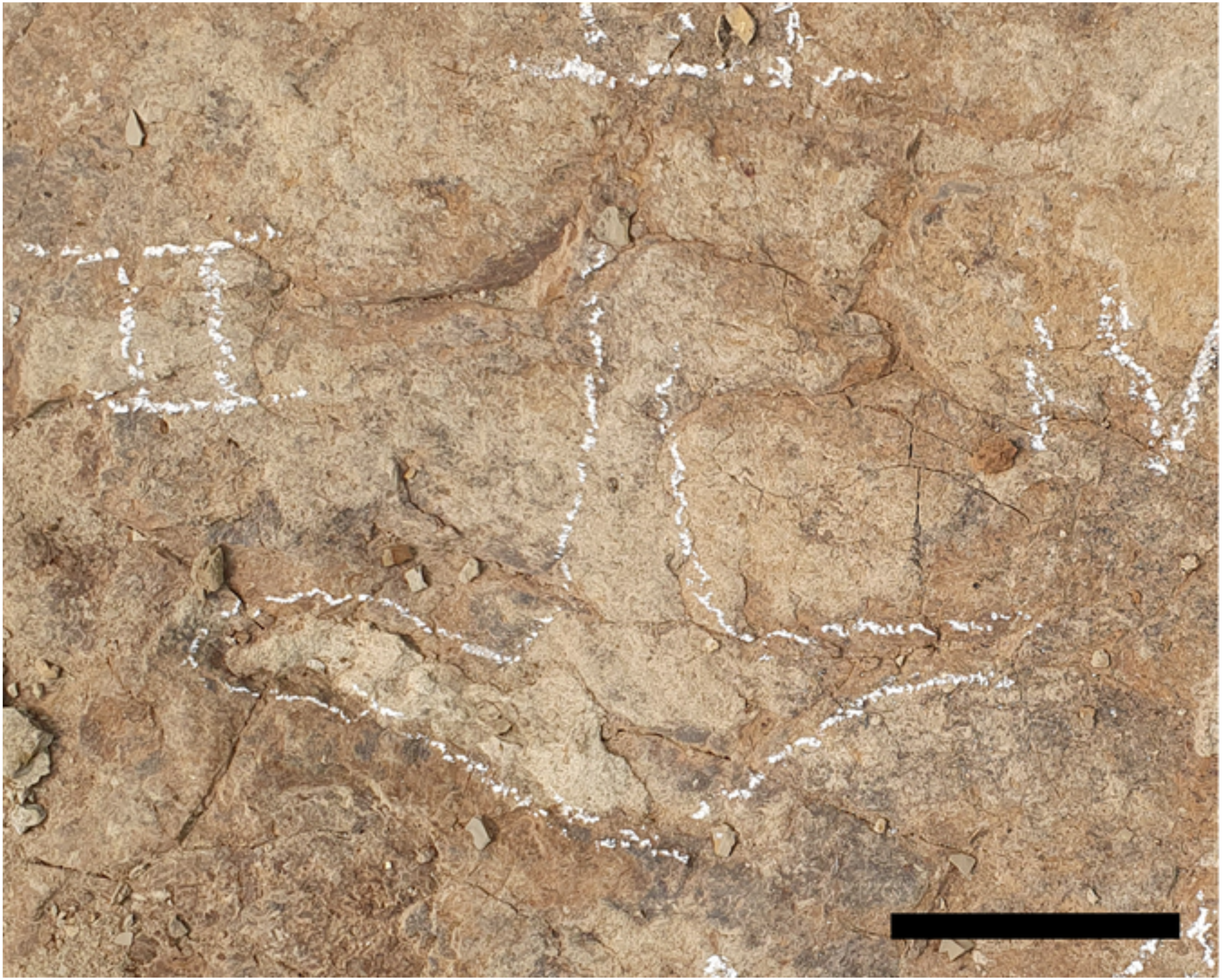

Fig 11

Click here to access/download;Figure;FIGURE_11_Cathedral.tif

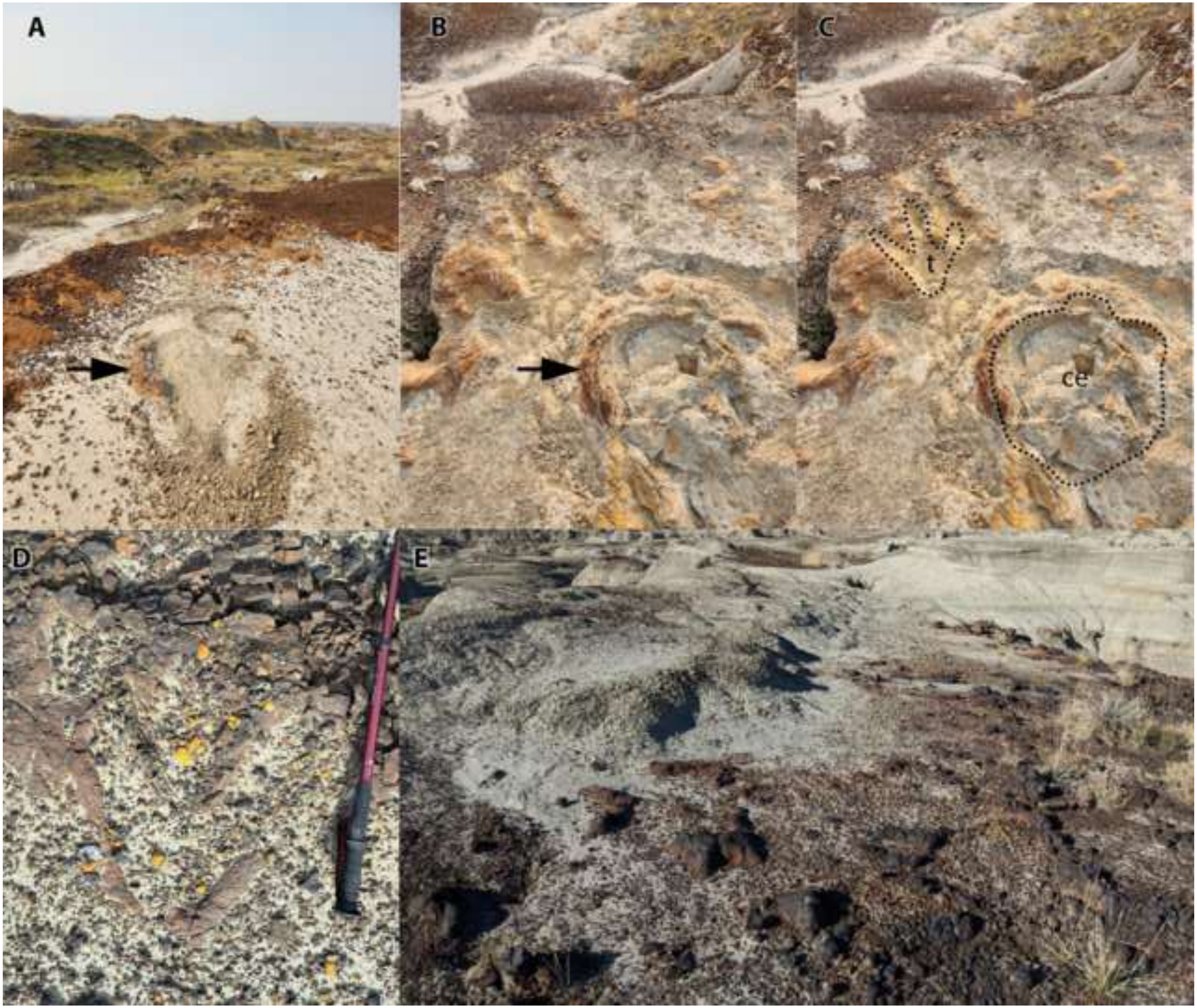

Supplement: Attachment — Submitted filename: PONE-D-25-02384.pdf [file pone.0324913.s002.pdf]
