# Supplementary material for: A ceratopsid-dominated tracksite from the Dinosaur Park Formation (Campanian) at Dinosaur Provincial Park, Alberta, Canada
Source: PLoS One. 2025 Jul 23;20(7):e0324913. doi: 10.1371/journal.pone.0324913 (PMC12286367; doi:10.1371/journal.pone.0324913)
Supplement: Attachment — Submitted filename: Response to reviewers.docx [file pone.0324913.s004.docx]

PONE-D-25-02384

**A ceratopsid-dominated tracksite from the Dinosaur Park Formation (Campanian) at Dinosaur Provincial Park, Alberta, Canada**

On behalf of my co-authors I would like to say that we are extremely grateful for the positive reviews and have endeavoured to make all of the suggested changes. We also note our responses to journal requirements. Please see specific responses to Reviewer 1 and Journal requirements below.

Response to Reviewer 1

Page 5, lines 88-90: This chapter is too short. Which were the methods you used, for example to measure the tracks?

Page 6, line 115: This should be shifted to the methods chapter

Page 6, line 125: Should also be shifted to the methods chapter

Page 6, line 135: methods

Page 7, line 141: methods

Page 7, line 157: methods chapter

**Response:** The intention here is for the Materials and Methods section to be divided up into subsections. The font sizes appear to be correct as per PLoS ONE requirements (Level 1: Materials and Methods = 18; Level 2 subheadings = 16) but we agree that this 2pt font size change is not the most obvious change between sections. As Excavation, Mapping, etc. are all subheadings under Materials and Methods we have made this clearer by adding “methods” to each of those subheadings.

Page 12, line 264: alternative verb?

**Response:** Thank you for catching this repetition, we have changed the wording to avoid this and it now reads “Three distinct types of invertebrate traces were present, all preserved as medium-grained sandstone infill with a host matrix.”

Page 21, lines 479-480: Are they probably a new ichnospecies?

**Response:** We appreciate that the morphology of the tyrannosaurid tracks discussed here could be interpreted as representative of a novel ichnotaxon. However, as demonstrated by Enriquez et al. (2021), there is substantial ontogenetic variation in tyrannosaurid tracks that has yet to be accounted for in the Dinosaur Park Formation. Until a better understanding of such changes is available (based on new discoveries - which, given the current rate of discovery, may not be in the too-distant future!) we prefer a conservative stance and leave the DPP tyrannosaur tracks in open nomenclature.

Page 22, Lines 484-486: Was it documented before via photogrammmetry?

**Response:** Yes, the trace was observed during the excavation and Fig. 10 is the photograph associated with the trace when it was found. We have now noted in the text that it was documented before photogrammetry.

Response to Journal requirements

**1.** When submitting your revision, we need you to address these additional requirements.

Please ensure that your manuscript meets PLOS ONE's style requirements, including those for file naming.

**Response:** Having thoroughly checked the documentation we believe our article meets all specified style requirements as per PLoS ONE instructions. The main issue appears to have been with Figure file naming which has now been corrected. If there are any that we have missed, please note exactly which files are causing problems and I will fix them right away.

**2.** For studies involving third-party data, we encourage authors to share any data specific to their analyses that they can legally distribute. PLOS recognizes, however, that authors may be using third-party data they do not have the rights to share. When third-party data cannot be publicly shared, authors must provide all information necessary for interested researchers to apply to gain access to the data. (https://journals.plos.org/plosone/s/data-availability#loc-acceptable-data-access-restrictions)

For any third-party data that the authors cannot legally distribute, they should include the following information in their Data Availability Statement upon submission:

1) A description of the data set and the third-party source

2) If applicable, verification of permission to use the data set

3) Confirmation of whether the authors received any special privileges in accessing the data that other researchers would not have

4) All necessary contact information others would need to apply to gain access to the data.

**Response:** Due to the sensitive nature of the site, which may be vulnerable to vandalism and is set to be further excavated in July 2025 we have removed the site coordinates from the Geological Setting section. We hope that the following data availability statement is acceptable but please let us know if you have any requested changes.

**Data Availability Statement:** All data necessary to replicate the analyses are presented in the manuscript and its Supporting Information files. The Skyline Tracksite is located within the Dinosaur Provincial Park Palaeontological Preserve. Due to the sensitivity of the site coordinates cannot be publicly shared but were provided to reviewers. Interested researchers may apply to gain access to the full site coordinate data, which are on file at the Royal Tyrrell Museum of Palaeontology, Drumheller, Alberta. Site number L2467. Please contact RTMP Collections: https://tyrrellmuseum.com/research/collections

**3.** Your ethics statement should only appear in the Methods section of your manuscript. If your ethics statement is written in any section besides the Methods, please move it to the Methods section and delete it from any other section. Please ensure that your ethics statement is included in your manuscript, as the ethics statement entered into the online submission form will not be published alongside your manuscript.

**Response:** Thank you for catching this. We have now removed the ethics part from the Acknowledgements section and moved it into the start of the Materials and Methods section.

**4.** We note that Figures 1 and 2 in your submission contain [map/satellite] images which may be copyrighted. All PLOS content is published under the Creative Commons Attribution License (CC BY 4.0), which means that the manuscript, images, and Supporting Information files will be freely available online, and any third party is permitted to access, download, copy, distribute, and use these materials in any way, even commercially, with proper attribution. For these reasons, we cannot publish previously copyrighted maps or satellite images created using proprietary data, such as Google software (Google Maps, Street View, and Earth). For more information, see our copyright guidelines: http://journals.plos.org/plosone/s/licenses-and-copyright.

We require you to either (1) present written permission from the copyright holder to publish these figures specifically under the CC BY 4.0 license, or (2) remove the figures from your submission:

**Response:** Figure 1 is made up of photographs taken by one of the lead authors (PRB) so we do not believe that copyright needs to be requested, but we have included a form noting that PRB is happy for the photos to be used as Fig 1 in the manuscript. Figure 2 is a set of site maps made in Adobe Illustrator (V 23.0.6) by study co-author CMB using the hand drawn maps that were created during the field work. Again we do not believe that there are any copyright issues here as no proprietary data was used, but we have included a form noting that CMB made these maps to be used as Fig. 2 in the manuscript.

**5.** Please review your reference list to ensure that it is complete and correct. If you have cited papers that have been retracted, please include the rationale for doing so in the manuscript text, or remove these references and replace them with relevant current references. Any changes to the reference list should be mentioned in the rebuttal letter that accompanies your revised manuscript. If you need to cite a retracted article, indicate the article’s retracted status in the References list and also include a citation and full reference for the retraction notice.

**Response:** We have gone through all of the references and corrected the doi’s to put them in the correct format, which appeared to be the problem here. Please see the tracked changes version of the manuscript for all changes to reference formatting. As far as we are aware none of the cited papers have been retracted, but if you have noted any please could you indicate which ones?
